# Supplementary figures and images for: Genome-wide identification and expression analysis of late embryogenesis abundant protein-encoding genes in rye (Secale cereale L.)
Source: PLoS One. 2021 Apr 8;16(4):e0249757. doi: 10.1371/journal.pone.0249757 (PMC8031920; doi:10.1371/journal.pone.0249757)

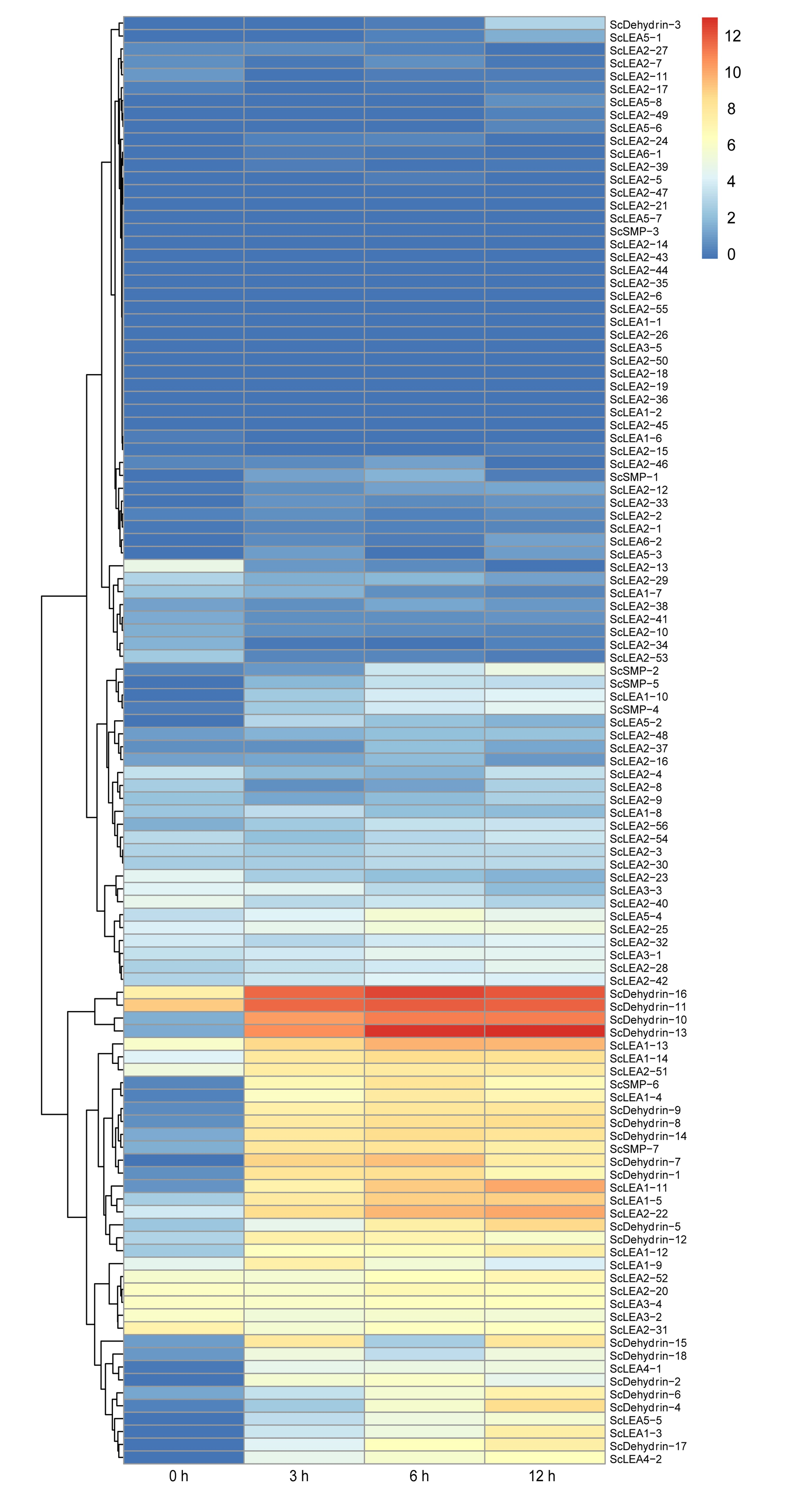

Supplement: S1 Fig — The heatmap was constructed using TBtools. The color scale on the right represents relative expression levels: red represents high level and blue represents low level. (TIF) [file pone.0249757.s001.tif]

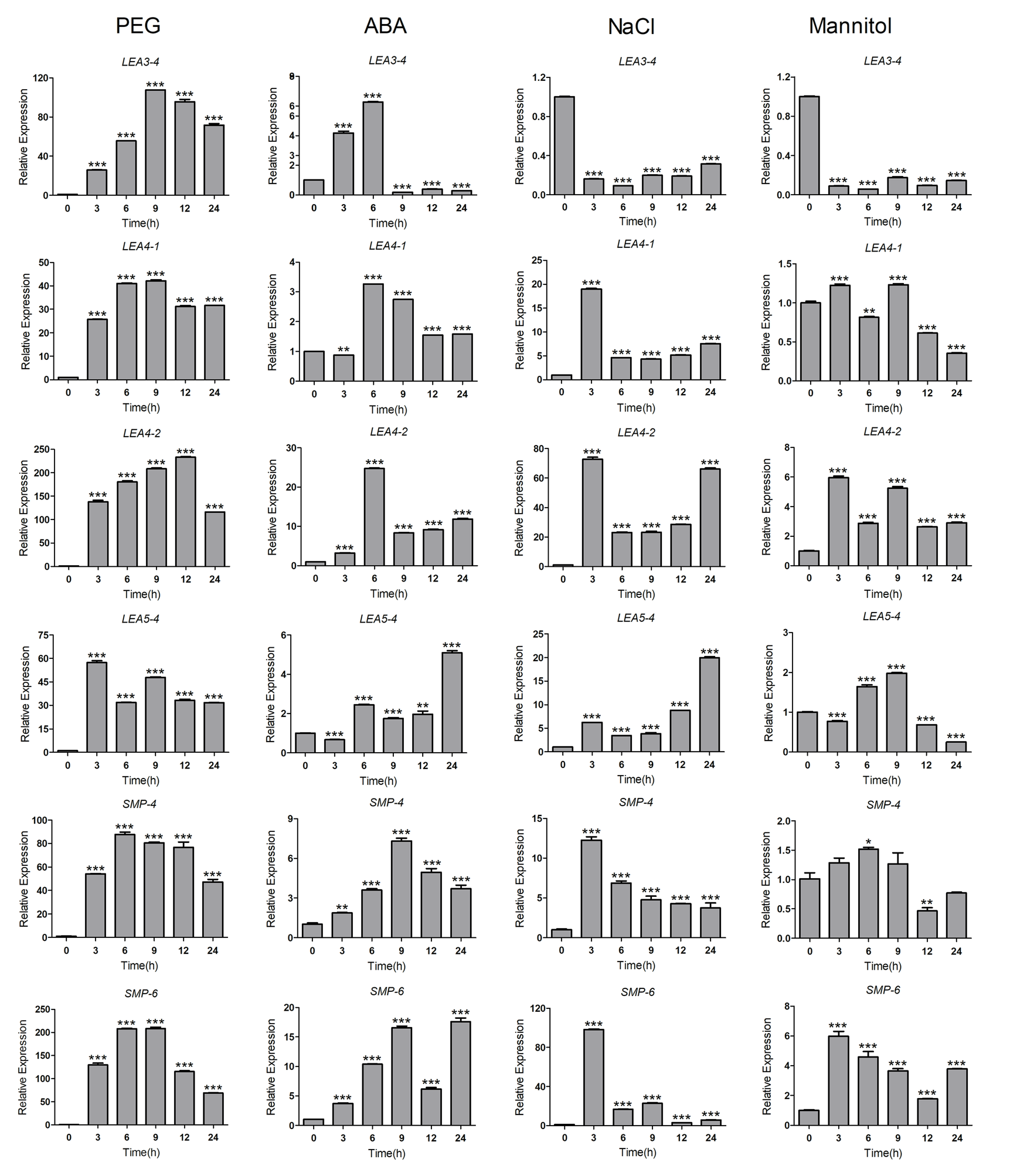

Supplement: S2 Fig — Ten-day-old seedling leaves were sampled after 0 h, 3 h, 6 h, 9 h, 12 h, and 24 h under 20% PEG6000, 100 μM ABA, 200 mM NaCl, or 100 mM mannitol. The values represent mean ± SEM of three replicates. The significant differences between data were calculated using Student’s t-test, and indicated with asterisks (*P <0.05, ** P<0.01, *** P<0.001). (TIF) [file pone.0249757.s002.tif]

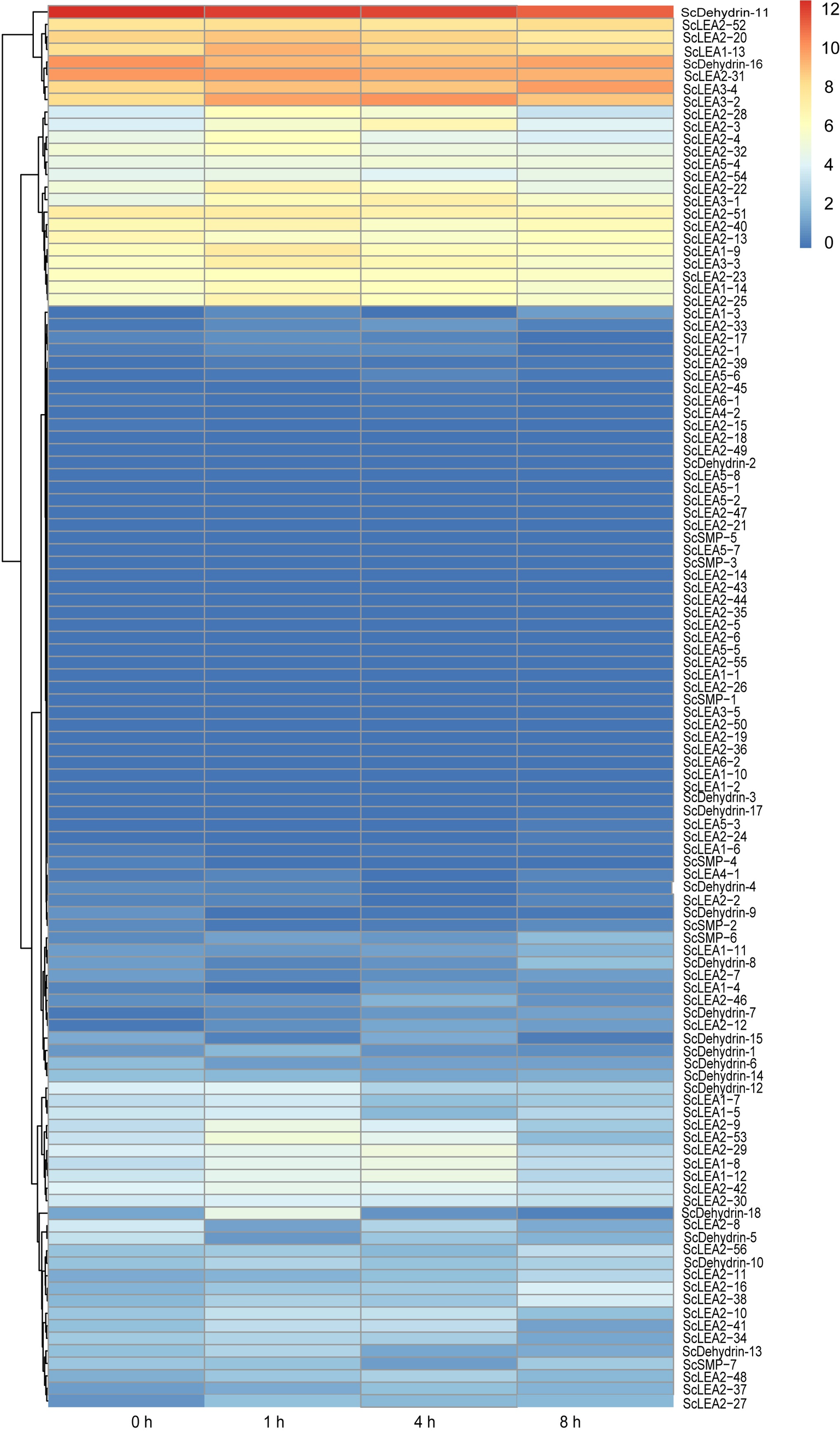

Supplement: S3 Fig — The heatmap was constructed using TBtools. The color scale on the right represents relative expression levels: red represents high level and blue represents low level. (TIF) [file pone.0249757.s003.tif]

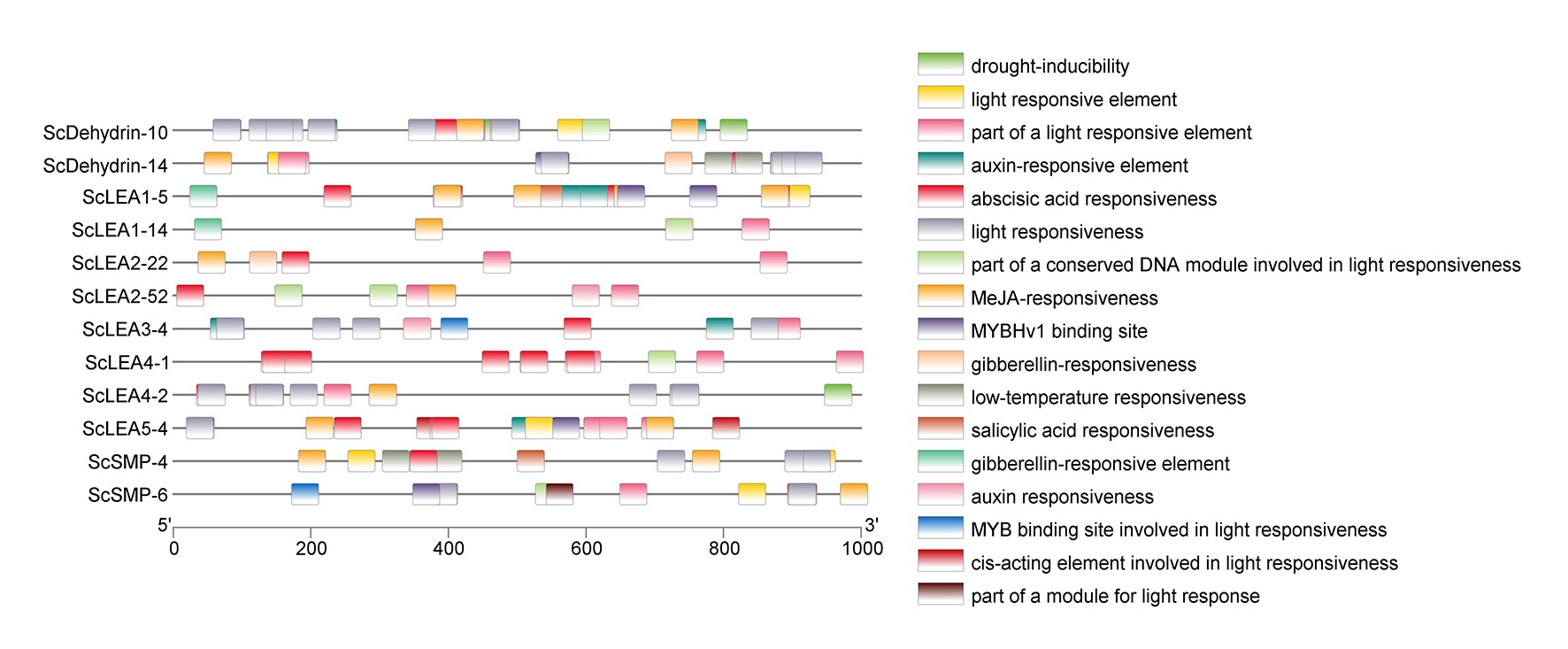

Supplement: S4 Fig — (TIF) [file pone.0249757.s004.tif]
