# Supplementary material for: Genome-wide identification and expression analysis of late embryogenesis abundant protein-encoding genes in rye (Secale cereale L.)
Source: PLoS One. 2021 Apr 8;16(4):e0249757. doi: 10.1371/journal.pone.0249757 (PMC8031920; doi:10.1371/journal.pone.0249757)
Supplement: Attachment — Submitted filename: PONE-D-21-02217_reviewer-edited.pdf [file pone.0249757.s009.pdf]

PLOS ONE

Genome-wide identification and expression analysis of late embryogenesis abundant protein-encoding genes in rye (Secale cereale L.)

--Manuscript Draft--

**Manuscript Number:**	PONE-D-21-02217
**Article Type:**	Research Article
**Full Title:**	Genome-wide identification and expression analysis of late embryogenesis abundant protein-encoding genes in rye (Secale cereale L.)
**Short Title:**	LEA genes in Secale cereale L.
**Corresponding Author:**	Lijian Wang<br>Henan Agricultural University<br>Zhengzhou, CHINA
**Keywords:**	late embryogenesis abundant proteins; expression profile; rye (Secale cereale L.); abiotic stress
**Abstract:**	Late embryogenesis abundant (LEA) proteins are members of a large and highly diverse family that play critical roles in protecting cells from abiotic stresses and maintaining plant growth and development. However, the identification and biological function of genes of Secale cereale L. LEA (ScLEA) have been rarely reported. In this study, we identified 112 ScLEA genes, which can be divided into eight groups and are evenly distributed on all rye chromosomes. Structure analysis revealed that members of the same group tend to be highly conserved. We identified 12 pairs of tandem duplication genes and 19 pairs of segmental duplication genes, which may be the expansion way of LEA gene family. Expression profiling analysis revealed obvious temporal and spatial specificity of ScLEA gene expression, with the highest expression levels observed in grains. According to qRT-PCR analysis, selected ScLEA genes were regulated by various abiotic stresses, especially PEG treatment, decreased temperature, and blue light. Taken together, our results provide a reference for further functional analysis and potential utilization of the ScLEA genes in improving the stress tolerance of crops.
**Order of Authors:**	Mengyue Ding
	Lijian Wang
	Weimin Zhan
	Guanghua Sun
	Xiaolin Jia
	Shizhan Chen
	Wusi Ding
	Jianping Yang
**Additional Information:**	
**Question**	**Response**
**Financial Disclosure**<br><br>Enter a financial disclosure statement that describes the sources of funding for the work included in this submission. Review the submission guidelines for detailed requirements. View published research articles from *PLOS ONE* for specific examples.	Jianping Yang.<br>the National Natural Science Foundation of China (31871709),<br>the Key Project of Beijing Natural Science Foundation (6151002),<br>the Technical Innovation Guidance Project of Henan Province (182106000050).

Powered by Editorial Manager® and ProduXion Manager® from Aries Systems Corporation

This statement is required for submission and **will appear in the published article** if the submission is accepted. Please make sure it is accurate.<br><br>**Unfunded studies**<br>Enter: *The author(s) received no specific funding for this work.*<br><br>**Funded studies**<br>Enter a statement with the following details:<br>• Initials of the authors who received each award<br>• Grant numbers awarded to each author<br>• The full name of each funder<br>• URL of each funder website<br>• Did the sponsors or funders play any role in the study design, data collection and analysis, decision to publish, or preparation of the manuscript?<br>• **NO** - Include this sentence at the end of your statement: *The funders had no role in study design, data collection and analysis, decision to publish, or preparation of the manuscript.*<br>• **YES** - Specify the role(s) played.<br><br>* typeset	
**Competing Interests**<br><br>Use the instructions below to enter a competing interest statement for this submission. On behalf of all authors, disclose any competing interests that could be perceived to bias this work—acknowledging all financial support and any other relevant financial or non-financial competing interests.<br><br>This statement is required for submission and **will appear in the published article** if the submission is accepted. Please make sure it is accurate and that any funding sources listed in your Funding Information later in the submission form are also declared in your Financial Disclosure statement.<br><br>View published research articles from *PLOS ONE* for specific examples.	The authors have declared that no competing interests exist.

**NO authors have competing interests**<br><br>Enter: *The authors have declared that no competing interests exist.*<br><br>**Authors with competing interests**<br><br>Enter competing interest details beginning with this statement:<br><br>*I have read the journal's policy and the authors of this manuscript have the following competing interests: [insert competing interests here]*<br><br>* typeset	
**Ethics Statement**<br><br>Enter an ethics statement for this submission. This statement is required if the study involved:<br><br>• Human participants<br>• Human specimens or tissue<br>• Vertebrate animals or cephalopods<br>• Vertebrate embryos or tissues<br>• Field research<br><br>Write "N/A" if the submission does not require an ethics statement.<br><br>General guidance is provided below. Consult the submission guidelines for detailed instructions. **Make sure that all information entered here is included in the Methods section of the manuscript.**	N/A

**Format for specific study types**<br><br>**Human Subject Research (involving human participants and/or tissue)**<br>• Give the name of the institutional review board or ethics committee that approved the study<br>• Include the approval number and/or a statement indicating approval of this research<br>• Indicate the form of consent obtained (written/oral) or the reason that consent was not obtained (e.g. the data were analyzed anonymously)<br><br>**Animal Research (involving vertebrate animals, embryos or tissues)**<br>• Provide the name of the Institutional Animal Care and Use Committee (IACUC) or other relevant ethics board that reviewed the study protocol, and indicate whether they approved this research or granted a formal waiver of ethical approval<br>• Include an approval number if one was obtained<br>• If the study involved *non-human primates*, add *additional details* about animal welfare and steps taken to ameliorate suffering<br>• If anesthesia, euthanasia, or any kind of animal sacrifice is part of the study, include briefly which substances and/or methods were applied<br><br>**Field Research**<br><br>Include the following details if this study involves the collection of plant, animal, or other materials from a natural setting:<br>• Field permit number<br>• Name of the institution or relevant body that granted permission	
**Data Availability**<br><br>Authors are required to make all data underlying the findings described fully available, without restriction, and from the time of publication. PLOS allows rare exceptions to address legal and ethical concerns. See the PLOS Data Policy and FAQ for detailed information.	Yes - all data are fully available without restriction

A Data Availability Statement describing where the data can be found is required at submission. Your answers to this question constitute the Data Availability Statement and **will be published in the article**, if accepted.<br><br>**Important:** Stating 'data available on request from the author' is not sufficient. If your data are only available upon request, select 'No' for the first question and explain your exceptional situation in the text box.<br><br>Do the authors confirm that all data underlying the findings described in their manuscript are fully available without restriction?	
**Describe where the data may be found in full sentences. If you are copying our sample text, replace any instances of XXX with the appropriate details.**<br><br>• If the data are **held or will be held in a public repository**, include URLs, accession numbers or DOIs. If this information will only be available after acceptance, indicate this by ticking the box below. For example: *All XXX files are available from the XXX database (accession number(s) XXX, XXX.).*<br>• If the data are all contained **within the manuscript and/or Supporting Information files**, enter the following: *All relevant data are within the manuscript and its Supporting Information files.*<br>• If neither of these applies but you are able to provide **details of access elsewhere**, with or without limitations, please do so. For example:<br><br>*Data cannot be shared publicly because of [XXX]. Data are available from the XXX Institutional Data Access / Ethics Committee (contact via XXX) for researchers who meet the criteria for access to confidential data.*<br><br>*The data underlying the results presented in the study are available from (include the name of the third party*	All relevant data are within the manuscript and its Supporting Information files.

and contact information or URL).<br>• This text is appropriate if the data are owned by a third party and authors do not have permission to share the data.<br><br>* typeset	
Additional data availability information:	

Powered by Editorial Manager® and ProduXion Manager® from Aries Systems Corporation

Click here to access/download;Manuscript;Ding.docx

Genome-wide identification and expression analysis of late embryogenesis abundant protein-encoding genes in rye (*Secale cereale* L.)

Mengyue Ding[1, †], Lijian Wang[1, 2*], Weimin Zhan[1], Guanghua Sun[1], Xiaolin Jia[1], Shizhan Chen[1], Wusi Ding[1], Jianping Yang[1*]

[1] College of Agronomy, Henan Agricultural University, Zhengzhou, 450000, China;

[2] Department of Criminal Science and Technology, Henan Police College, Zhengzhou, 450000, China

[†] These authors contributed equally to this work.

[*] jpyang@henau.edu.cn (JY); wanglijian2012@163.com (LW);

Short title: LEA genes in *Secale cereale* L.

Abstract: Late embryogenesis abundant (LEA) proteins are members of a large and highly diverse family that play critical roles in protecting cells from abiotic stresses and maintaining plant growth and development. However, the identification and biological function of genes of *Secale cereale* ~~L.~~ *LEA* (*ScLEA*) have been rarely reported. In this study, we identified 112 *ScLEA* genes, which can be divided into eight groups and are evenly distributed on all rye chromosomes. Structure analysis revealed that members of the same group tend to be highly conserved. We identified 12 pairs of tandem duplication genes and 19 pairs of segmental duplication genes, which may be ~~the~~ expansion way of *LEA* gene family. Expression profiling analysis revealed obvious temporal and spatial specificity of *ScLEA* gene expression, with the highest expression levels observed in grains. According to qRT-PCR analysis, selected *ScLEA* genes were regulated by various abiotic stresses, especially PEG treatment, decreased temperature, and blue light. Taken together, our results provide a reference for further functional analysis and potential utilization of the *ScLEA* genes in improving ~~the~~ stress tolerance of crops.

Keywords: late embryogenesis abundant proteins; expression profile; ~~rye (~~*Secale cereale* L.~~)~~; abiotic stress.

Introduction

Plants are affected by a variety of biotic and abiotic stresses during their lifetime due to their inability to escape harmful ~~external~~ environmental conditions. Late embryogenesis abundant (LEA) proteins are highly hydrophilic proteins with antioxidant and ion binding properties that function in membrane and protein stabilization, osmotic regulation, and as a protective buffer against dehydration [1]. Thus, they play important roles in protecting cells from abiotic stress, and in normal growth and development. LEA proteins were first isolated from cotyledons of cotton, at ~~the~~ late embryonic development stage, [2], and in recent decades, have also been found in species ~~ranging~~ from algae to angiosperms, such as cyanobacteria [3], *Arabidopsis thaliana* [4], rice (*Oryza sativa* L.) [5], wheat (*Triticum aestivum* L.) [6], maize (*Zea mays* L.) [7], and in prokaryotes and invertebrates, such as rotifers [8, 9]. In plants, *LEA* genes are highly expressed at the late stage of seed maturation [2] and LEA proteins accumulate in roots, stems, and other organs throughout the plant growth ~~phase~~ [10].

In higher plants, based on the similarity of amino acid sequences and differences in conserved domains, the LEA family is generally divided into eight groups: LEA1, LEA2, LEA3, LEA4, LEA5, LEA6, Dehydrin, and seed maturation protein (SMP) [11]. In recent studies abscisic acid (ABA) stress ripening (ASR) proteins were classified as LEA7 group proteins [12, 13], extending the LEA family to nine groups. The division of LEA family members varies among species [14]. Most *LEA* genes encode small proteins with molecular weights ranging from 10 to 30 kDa, and they are predominantly composed of a repeating arrangement of hydrophilic amino acids that form a highly hydrophilic structure [15]. Each group of LEA proteins has its own unique conserved motif [16]. For example, LEA1 proteins contain a 20-amino-acid motif (GGETRKEQLGEEGYREMGRK) [17]; Dehydrin proteins contain a motif called the K-segment (EKKGIMDKIKEKLPG) [18, 19]; LEA4 proteins have a conserved domain of 11-amino-acid sequences (TAQAAKEKAGE) [18]. These conserved sequences (motifs) have been preserved through long-term evolution, and

play an important role in plant responses to environmental stress.

Previous studies have shown that LEA proteins are ubiquitous and localized in the cytoplasm, nucleus, chloroplast, mitochondria, and endoplasmic reticulum [20]. Thus, not all LEA proteins are localized the same part of the cell, and that their particular functions depend on their intra-cellular locations. The expression of LEA proteins is often induced by abiotic stresses such as drought, heat, cold, and exogenous hormone at different development stages and tissues of plants [1, 4]. Moreover, overexpression of *LEA* genes can improve stress tolerance of transgenic plants. For example, overexpression of the barley (*Hordeum vulgare* L.) *HVA1* gene promoted drought and salt stress tolerance in rice [21] and wheat [22]. Overexpression of the *Arabidopsis* Dehydrins group proteins altered pathogenesis-related protein expression and activated defense response [23]. The above studies demonstrate that *LEA* genes could potentially be used to improve the abiotic stress tolerance of crops.

Rye (*Secale cereale* L.) is a diploid Triticeae species closely related to wheat and barley, and an important crop for feed and food [24]. Rye has excellent disease and stress resistance, and it has been used as a source to improve wheat resistance to pathogens in the past few decades [25]. Rye-wheat chromosome translocation has been widely used in wheat breeding around the world, and translocation lines have notably increased yield [26, 27]. Studies on LEA proteins in wheat and barley have been reported [6, 22]. However, the distribution and function of LEA proteins in rye have been rarely reported [28]. In this study, we identified 112 ~~*Secale cereale* L. *LEA* (~~*ScLEA*~~)~~ genes based on rye genome sequence. The phylogenetic analysis, structural characterizations, evolutionary relationships, and expression profiles of *ScLEA* genes were systematically analyzed. These results will contribute to our understanding of the LEA family in rye and to the utilization of these *LEA* genes in homologous or heterologous systems.

Materials and methods

Plant materials, sample preparation, and stress treatments

Rye (Weining) plants were grown under greenhouse conditions with a temperature of 18°C and a 16 h light/8 h dark photoperiod. For RNA sequencing (RNA-seq), root, stem, leaf, and spikelet samples were collected at the heading stage. The developing grain samples were collected at 10, 20, 30, and 40 days after anthesis (DAA). Leaves were collected from plants cultivated under normal conditions (CK) and cold conditions (-10°C) for 0 h, 1 h, 4 h, and 8 h. For drought treatment, the leaves were dried between folds of tissue paper for 0 h, 3 h, 6 h, and 12 h. Three biological replicates were collected for each sample.

For abiotic stress treatments, ~~the~~ rye ~~(Weining)~~ seedlings were grown in hydroponics with a temperature (18°C) for 10 days, and then treated with 20% (w/v) PEG6000, 200 mM NaCl, 100 mM mannitol, 100 μM ABA, or temperatures of 0°C or 4°C. Leaves were collected after 0 h, 3 h, 6 h, 9 h, 12 h, and 24 h. Leaf samples were treated with sterile water and similarly collected as a control. For different light treatments, seedlings were grown in the dark for seven days and subsequently transferred to far-red (FR) light (5 μmol·m^{-2}·s^{-1}), red (R) light (17.56 μmol·m^{-2}·s^{-1}), blue (B) light (13 μmol·m^{-2}·s^{-1}), or White (W) light (85 μmol·m^{-2}·s^{-1}) for 4 h. For each sample, three leaves treated in parallel represented three biological replicates. All treated tissue samples were stored at -80℃ for subsequent analysis.

Identification of *LEA* genes in the rye genome

The nucleotide and protein sequences, as well as the gene annotation files, were downloaded from the rye genome database (http://pgsb.helmholtz-muenchen.de/plant/rye/index.jsp). The HMM profiles (LEA1: PF03760, LEA2: PF03168, LEA3: PF03242, LEA4: PF02987, LEA5: PF00477, LEA6: PF10714, Dehydrin: PF00257, SMP: PF04927) were downloaded from the Pfam database (http://pfam.sanger.ac.uk/). Then HMMER 3.0 was used to search the encoded protein sequences with the default parameters and a filter threshold of 0.01. In addition, the obtained amino acid sequences were used as queries in searches

against both the CDD database (https://www.ncbi.nlm.nih.gov/cdd/), and SMART database (http://smart.embl-heidelberg.de/), and the repeated or non-LEA domain sequences were eliminated manually. Finally, the identified rye LEA proteins were named according to group and gene ID order. The physical and chemical properties of ScLEA proteins were analyzed using the ProtParam online tool (https://web.expasy.org/protparam/).

Phylogenetic analysis of LEA proteins in rye

To investigate the evolutionary relationships of LEA proteins in rye, multiple alignments of the full-length protein sequences were performed using MAFFT [29] (http://mafft.cbrc.jp/alignment/software/) with the default parameters. A phylogenetic tree was constructed based on these alignments using the Neighbor-Joining (NJ) method with a bootstrap test of 1000 replicates for assessing internal clade reliability using MEGA X software [30]. The Evolview website (https://www.evolgenius.info/evolview/#login) was used to modify the phylogenetic tree.

Analysis of gene structures and conserved domains of *LEA* genes in rye

The exon-intron structures of the *ScLEA* family genes were determined by aligning the coding sequences with the corresponding genomic sequences, and visualized using the online software GSDS (http://gsds.gao-lab.org/index.php). Conserved domains of the ScLEA proteins were predicted using MEME (http://meme-suite.org/index.html).

Distribution of *LEA* genes on rye chromosomes

ScLEA genes were mapped on rye chromosomes according to the positional information from the rye genome annotation database, and the chromosome physical location map was displayed using Mapchart [31] software. The Multiple Collinearity Scan toolkit (MCScanX) [32] with default parameters was used to analyze gene duplication events.

Expression profile analysis of rye *LEA* genes

The transcripts of the datasets were aligned, followed by merging and removal of redundant sequences using Hisat (http://ccb.jhu.edu/software/hisat/index.shtml)

(version 2.0.4) and Stringtie (http://ccb.jhu.edu/software/stringtie/) (version 1.2.3). The software featureCounts [33] was used to calculate the number of reads and normalized gene expression levels. Expression heatmaps were generated using TBtools software [34].

RNA extraction and gene expression analysis

Total RNA was extracted from the leaves of rye using Eastep® Super RNA Kit (Promega). The GoScript™ Reverse Transcriptase Kit (Promega) was used to reverse-transcribe RNA into cDNA. Quantitative real-time PCR (qRT-PCR) was carried out ~~with~~ the Roche Lightcyler® 480 instrument using SYBR Green (TaKaRa). The *ScActin* gene was used as an internal control. The thermal cycler program used was 95℃ for 10 min, and followed by 45 cycles of 95℃ for 10 s, 60℃ for 10 s, and 72℃ for 20 s. Each reaction was performed in three technical replicates, and the data from qRT-PCR were calculated using the $2^{-\Delta\Delta Ct}$ method [35]. Sequences of the primers used in this study are shown in Table S1.

Cis-acting element analysis

The Plant CARE website (http://bioinformatics.psb.ugent.be/webtools/plantcare/html/) was used to predict *cis*-acting elements in the regions 1000 bp upstream of the gene start codon and a diagram was visualized using TBtools software [34].

Results

Genome-wide identification and phylogenetic analysis of *ScLEA* genes

Based on genome-wide blast searches, 112 *ScLEA* genes were identified from the rye genome database. The identity of the sequences was verified by checking for the presence of conserved domains using the CDD and SMART tools. These 112 genes were divided into eight groups (LEA1–LEA6, Dehydrin, and SMP) based on the phylogenetic analysis (Figure 1). The largest group was LEA2, which contained 56 members, while the smallest groups, LEA4 and LEA6, had only two members each. Groups Dehydrin, LEA1, LEA5, SMP, and LEA3 contained 18, 14, 8, 7, and 5 members, respectively.

The physical and chemical parameters of the 112 ScLEA proteins were calculated

using the ProtParam online tool (Table S2). The smallest ScLEA protein had 78 amino acid residues with a molecular weight of 8.24 kDa (ScLEA5-8) and the largest had 965 amino acid residues with a molecular weight of 99.64 kDa (ScDehydrin-12). The isoelectric point (pI) values ranged from 4.22 (ScSMP-6) to 11.07 (ScLEA2-17), with an average of 8.37. Most LEA proteins have low hydrophobicity and a large net charge [4, 36, 37], which allow them to be "completely or partially disordered", these proteins may form molecular chaperones that contribute to the protection of plants from desiccation [38, 39]. Consistent with this, grand average of hydropathicity (GRAVY) index analysis indicated that most of the ScLEA proteins were hydrophilic. However, the ScLEA2 group was atypical with 67.8% of proteins were predicted as hydrophobic. The aliphatic index reflects the thermal stability of the protein.

Phylogenetic analysis (Figure 1) revealed two major clades of the ScLEA family. The LEA2 group proteins were clustered in one clade, while the proteins in the other seven groups formed another clade. According to the evolutionary relationships, one clade including the Dehydrin group (18 numbers) and LEA5 group proteins (8) was closely related to the LEA4 (2), LEA1 (14), and SMP (7) groups, but distant from the LEA6 (2) and LEA3 (5) groups.

Structural characterization of *ScLEA* genes

The structure of genes is important for determining their expression and function. Structural analysis revealed that the *ScLEA* genes contain few introns; 42% of *ScLEA* genes have one intron, 5.4% have two introns, 0.8% have three introns, and 51.8% possess no introns (Figure 2). There were four intron-free genes and fourteen single-intron genes in the ScDehydrin group. In the ScLEA1 group, seven genes contained no intron, six genes contained one intron, and only one gene contained two introns (*ScLEA1-7*). In the ScLEA2 group, there were 41 intron-free genes, nine single-intron genes, five double-intron genes, and a triple-intron gene (*ScLEA2-37*). There were four free-intron genes and one single-intron gene in the ScLEA3 group. All genes in the ScLEA4, ScLEA5, and ScSMP groups had one intron, and all genes in the ScLEA6 group had no introns. *ScLEA* genes in the same group had similar numbers of exons and introns, indicating that members of the same family tend to be

highly conserved.

To elucidate the similarity and diversity of protein domains, each group was separately submitted to MEME for structure analysis. The results showed that each group contained one or more conserved LEA domains (Figure 2). Moreover, members of the same group were more similar to each other than to members of other groups, indicating that each group of ScLEA proteins may have specific functions.

Chromosomal locations and gene duplication of *ScLEA* genes

The distribution of *ScLEA* genes was determined by mapping their positions on the rye chromosomes. The 112 *ScLEA* genes were distributed on all seven chromosomes, and their distribution was nearly even among different chromosomes (Figure 3). Chromosome 5 had the most *ScLEA* genes (17.8%, 20 genes), and chromosome 7 had the fewest (12.5%, 14 genes). Chromosomes 1, 2, 3 and 6 each contained 15 genes. Moreover, genes of the largest group may be distributed across all chromosomes to ensure maximum functionalization [6]. ~~For example, members of ScLEA2 group are distributed on all chromosomes.~~ However, other LEA groups have a limited distribution and are mainly found on specific chromosomes.

High-density *ScLEA* gene clusters were identified in certain chromosomal regions. According to Holub [40], we defined a chromosomal region of 200 kb containing two or more genes as a tandem duplicated event. Twenty-eight *ScLEA* genes (*ScLEA5-1/2*, *ScLEA5-4/5*, *ScLEA2-9/10*, *ScLEA2-13/14/15*, *ScLEA2-18/19*, *ScLEA1-2/3*, *ScLEA3-2/3*, *ScLEA2-31/32*, *ScSMP-1/2*, *ScDehydrin-7/8/9/10*, *ScDehydrin-12/13*, and *ScDehydrin-14/15/16*) were clustered into 12 tandem duplicated regions on chromosomes 1, 2, 3, 4, 5, and 6 (Table S3). Chromosome 2 had four clusters, indicating that it is a hot spot for *ScLEA* genes. In addition to the tandem duplication events, 19 segmental duplication events with 27 *ScLEA* genes were also identified using BLASTP and MCScanX (Figure 4, Table S4). These results indicated that some *ScLEA* genes ~~were~~ possibly generated by tandem duplication and segmental duplication events.

The expression profiles of *ScLEA* genes

RNA-seq data for ~~rye (Weining) different tissues~~ (root, stem, leaf, spikelet, and grain)

were used to explore the potential biological functions of *ScLEA* genes in growth and development. Expression profiles showed that most *ScLEA* genes were expressed with obvious spatial specificity (Figure 5). *ScLEA2-26* and *ScLEA2-43* were only expressed in spikelets, while *ScLEA2-3*, *ScLEA2-4*, *ScLEA2-27*, *ScLEA2-28*, and *ScLEA2-29* were only expressed in roots. The expression levels of *ScLEA1-3*, *ScLEA1-11*, *ScLEA2-22*, *ScDehydrin-5*, and *ScDehydrin-13* increased continuously during grain development, and reached a maximum at 40 DAA, indicating that these genes may be involved in grain grouting. ~~In addition,~~ *ScDehydrin-11*, *ScLEA2-20*, *ScLEA2-31*, and *ScLEA2-51* were highly expressed in different tissues, indicating that they are important at all stages of rye growth and development.

One characteristic of LEA proteins is induction of expression under environmental stresses such as drought and ABA [5, 6]. Therefore, we analyzed the expression profiles of 112 *ScLEA* genes after drought treatment (Figure S1). We found that the expression levels of *ScDehydrin-10*, *ScDehydrin-11*, *ScDehydrin-13*, and *ScDehydrin-16* increased the most, followed by *ScLEA1-11*, *ScLEA1-13*, and *ScLEA1-14*; thus, these genes may play major roles in drought stress response. The responses of ScLEA2 group genes were diverse due to its large number of members. For example, *ScLEA2-22* and *ScLEA2-31* respectively showed a significant upregulation and downregulation to drought stress. The responses of the ScLEA5 and ScLEA6 group genes were not obvious, with only the induction of *ScLEA5-5* being prominent.

The responses of *ScLEA* genes to abiotic stress

To verify the potential roles of *ScLEA* genes in abiotic stress, we randomly selected 12 *ScLEA* genes from eight groups, and their expression in rye leaves after treatment with PEG, ABA, NaCl, or mannitol after 0 h, 3 h, 6 h, 9 h, 12 h, and 24 h were analyzed by qRT-PCR. Similar to the results of RNA-seq under drought treatment (Figure S1), the results of qRT-PCR showed that the expression of these *ScLEA* genes was induced by different stress treatments (~~Figure 6, Figure 2~~). Among all treatments, *ScLEA* genes showed the most intense response to PEG, and compared with the control group, the level of *ScLEA* gene upregulation hundreds of times higher (except

for *ScLEA2-22* and *ScLEA2-52*) and lasted for a long time (12–24 h). The 12 *ScLEA* genes did not show the same response to ABA treatment, and even some genes were inhibited (*ScLAE2-22* and *ScLEA3-4*). The expression levels of most genes reached a maximum within 3 h after NaCl treatment, and the subsequent changes were quite different between genes. For some genes, expression was maintained at a continuously high level (*ScDehydrin-14*, *ScLEA1-5*, *ScLEA4-2*, and *ScSMP-4*), while the expression levels of others decreased rapidly (*ScDehydrin-10* and *ScSMP-6*). Under mannitol treatment, only the expression of *ScLEA*2-22 was significantly upregulated and the expression of some genes was inhibited (*ScLEA2-52* and *ScLEA3-4*).

The results of qRT-PCR analysis showed that genes of the ScLEA1, ScLEA2, ScDehydrin, and ScSMP groups were highly sensitive to the four treatments, with significant upregulation of gene expression. Interestingly, the expression of genes in the ScLEA3 group decreased under three treatments (ABA, NaCl, and mannitol), indicating that these treatments repressed gene expression. The above results showed that the gene expression patterns were different under the four treatments, and that the expression levels of genes in each group were also significantly different under the same treatment. The expression of these genes validates the RNA-seq results and also reflects the diversity of *ScLEA* gene functions in response to abiotic stress.

The response of *ScLEA* genes to cold stress

RNA-seq data analysis showed that *ScLEA* genes were responsive to different durations of low temperature stress treatment (Figure S3). Combined with the cold tolerance of rye [41], we analyzed the expression patterns of the same 12 genes under cold stress. *ScLEA* genes expression in rye leaves incubated at 4℃ and 0℃ for 0 h, 3 h, 6 h, 9 h, 12 h, and 24 h were analyzed by qRT-PCR. The qRT-PCR results showed that most of genes were sensitive to cold stress and showed different degrees of response (Figure 7). The results showed that *ScLEA* genes expression increased with increasing duration of cold treatment, and expression was significantly higher than that of the control by 12 h. The responses of *ScLEA* genes to 0℃ treatment were significantly stronger than those to 4℃ treatment at almost all treatment time points. Among the 12

genes, *ScDehydrin-10*, *ScLEA2-22*, and *ScSMP-6* were strongly responsive to low temperature, and their expression levels were upregulated to hundreds of times compared with the control (0 h). Similar to PEG, ABA, NaCl, and mannitol treatment, the gene expression of *ScLEA2-52* and *ScLEA3-4* to low temperature were inhibited at some time points, suggesting that these genes may be involved in other stress responses, such as biotic stress, or plant growth and development processes. In conclusion, the response of *ScLEA* genes to cold stress reflects the tolerance of rye to low temperatures.

The response of *ScLEA* genes to photon switching

To elucidate the possible regulatory mechanisms of *ScLEA* genes involved in environmental stress, we analyzed the stress-responsive elements in the promoter regions of 12 *ScLEA* genes (Figure S4). The ABRE, which is involved in ABA responsiveness, the LTRE, which responds to low temperature, the MBS (MYB binding site) element, which is involved in drought response, the G-box, Box 4 and GT1-motif, which participate in light responsiveness. The ABRE and G-box elements were found in most of the 12 genes, and auxin, salicylic acid, and jasmonic acid responsive elements were also identified.

~~In this study, the~~ responses of 12 *ScLEA* genes to PEG, ABA, NaCl, mannitol, and low temperature were confirmed. The role of *LEA* genes in photon switching response has rarely been reported. To verify whether the *ScLEA* genes respond to photon switching, the expression of *ScLEA* genes in rye leaves treated with FR, R, B and W light for 4 h were analyzed by qRT-PCR (Figure 8). Most of these *ScLEA* genes (*ScLEA1-5*, *ScLEA1-14*, *ScLEA2-22*, *ScLEA2-52*, *ScLEA4-1*, *ScLEA4-2*, *ScDehydrin-10*, and *ScDehydrin-14*) showed obvious responses to blue light. Compared with W light, *ScSMP-4*, *ScLEA4-1*, and *ScLEA4-2* showed a stronger response to R light. The results showed that *ScLEA* genes respond to different types of light, and that the degree of response to the four types of light varied greatly. Thus, plants may express various genes including *LEA* in response to stress.

Discussion

Identification and analysis of rye *LEA* genes

LEA genes have been reported in many species; however, the genomic identification and annotation of *LEA* genes has not been reported in rye. In this study, 112 *ScLEA* genes were identified, and were divided into eight groups. LEA2, the largest group in the rye family, ~~accounted~~ for about 50% of the total number of family members. The LEA2 group of wheat is also the largest, accounting for 159 of all 281 *LEA* genes [6]. However, the largest group in rice [5] and *Brassica napus* [42] is Dehydrin and LEA4, respectively. The distributions of *LEA* family members in different species indicate that *LEA* genes play unique roles in stress response and regulation of plant growth and development in different species. These differences also suggest that the *LEA* family genes may have evolved independently after the divergence of these species.

Normally, the diversity of structures and conserved domains caused the evolution of multigene families [43]. Our analysis revealed that the majority of LEA proteins in the same group shared similar domains, suggesting that these conserved domains may play crucial roles in group-specific functions. However, high divergence was also found in the structures between different groups. Identical results were reported earlier for LEA proteins in *Arabidopsis* [44], rice [5], and tomato (*Solanum lycopersicon* L.) [45]. Rye has the largest genome (~7.9 Gbp) among all diploid species in Triticeae, with more than 90% repetitive sequences [46]. Gene duplication is considered to be one of the primary driving forces in the evolution of genomes and genetic systems [47]. Twenty-eight *ScLEA* genes were clustered into 12 tandem duplication blocks, and 27 *ScLEA* genes were clustered into 19 segmental duplication blocks in rye. These results indicate that members of the same group may have originated via gene duplication, and that different groups may have evolved from different ancestral genes with various domain structures [48].

Expression analysis and functional prediction of rye *LEA* genes

Analysis of expression profiles in different tissues revealed obvious temporal and spatial specificity in *ScLEA* genes expression, with the highest expression for most genes mainly observed in grains (Figure 5). The expression levels of *LEA* genes in *Arabidopsis* [4] and wheat [49] were also reported to be generally higher in seeds.

Transcriptome data for rye subjected to drought stress showed that, except for *ScDehydrin-3*, all Dehydrin group genes were responsive to drought treatment (Figure S1). Dehydrin group genes were also specifically and highly expressed in grains (Figure 5). These results showed that the expression levels of Dehydrin group genes are upregulated during seed maturation, suggesting that the expression of these genes might be induced by dehydration at the later stage of seed maturation [49]. The correlation between LEA protein and desiccation tolerance had been confirmed in many orthodox seeds [21, 50]. During the mature dehydration of seeds, the Dehydrin protein will be present as space filler in cells, maintaining the dissolved character of cell fluid to avoid damage to cell architecture [15]. The molecular function of Dehydrin proteins in the process of seed maturation will provide new ideas for the genetic improvement of crops.

Light affects plant growth and developmental processes including seed germination, dormancy, circadian rhythms, flower induction, plant architecture, and shade avoidance [51]. Studies have found that LEA protein expression in *Escherichia coli* promotes tolerance to UV radiation [52, 53]. At present, studies of LEA proteins in plants generally focus on drought, salt, plant hormone, and low temperature stresses, including UV radiation stress, but few studies have been conducted on the response to different light qualities. Our results showed that *ScLEA* genes responded to FR, R, B, and W light, and that the degree of response to the four light treatments varied greatly. Thus, *ScLEA* genes may play certain roles in light responses, and those finding may provide a new perspective for the future study of new functions of ScLEA proteins. Rye is mainly grown in northern Europe and North Africa [54], and the mountainous or colder northern parts of China. Most of the regions experience a wide range of temperatures, altitudes, rainfall, and light levels. Therefore, the analysis of different stress treatments in this study lays ~~the~~ foundation for exploring the function of LEA proteins in improving the stress tolerance of rye.

Cis-acting elements involved in the mediation of rye _LEA_ gene expression

Plants frequently adapt to environmental stresses by regulating the rate of transcription [55]. *Cis*-acting elements of genes are binding sites for transcription

factors that activate or repress transcription [56]. Some promoter elements, such as the TATA, GC, and CCAAT boxes, are common to many genes. In response to abiotic stresses such as heat shock and hormones, a large number of specific elements are also involved in transcriptional regulation [56]. In *Arabidopsis*, most genes encoding LEA proteins were found to have ABRE and LTRE elements in their promoters and many genes containing these elements were induced by ABA, cold, or drought treatment [4]. In this study, we also found ABRE and LTRE elements in the *ScLEA* promoters (Figure S4). The results of qRT-PCR showed that the expression of 12 *ScLEA* genes was induced by ABA treatment and low temperature, and that the expression levels were significantly upregulated with a decrease of temperature. Moreover, G-box, Box 4, and GT1-motifs were also found in the *ScLEA* promoter, and *ScLEA* genes responded to light. Therefore, *cis*-acting elements that may influence the expression of *ScLEA* should also be investigated when analyzing the role of *ScLEA* genes under various stresses.

Conclusion

LEAs are important proteins that respond to biotic and abiotic stresses and have been described in numerous plants. In this study, a total of 112 *ScLEA* genes were identified in rye and classified into eight groups. All *ScLEA* genes contained few or no introns, and all encoded proteins containing the conserved LEA domain. *ScLEA* genes were distributed on all rye chromosomes with some clustering. By analyzing promoter elements in combination with RNA-seq data and qRT-PCR results, we showed that *LEA* genes in rye are strongly responsive to abiotic stresses such as drought, low temperature, light quality, ABA, and NaCl. These results lay a foundation for further investigating the functions of LEA proteins and their potential use in genetic improvement of crops.

Funding: This research was supported by the National Natural Science Foundation of China (31871709), the Key Project of Beijing Natural Science Foundation (6151002), and the Technical Innovation Guidance Project of Henan Province (182106000050).

Author Contributions

Conceptualization: Jianping Yang, Lijian Wang.

Funding acquisition: Jianping Yang.

Formal analysis: Mengyue Ding, Lijian Wang, Weimin Zhan.

Methodology: Guanghua Sun, Xiaolin Jia, Shizhan Chen, Wusi Ding.

Software: Weimin Zhan.

Investigation: Guanghua Sun, Xiaolin Jia.

Resources: Mengyue Ding, Weimin Zhan.

Writing - original draft preparation: Mengyue Ding, Lijian Wang.

Writing - review and editing: Jianping Yang.

All authors have read and agreed to the published version of the manuscript.

Competing interests: The authors declare no conflicts of interest to this work.

Reference

1. Chen Y, Li C, Zhang B, Yi J, Yang Y, Kong C, et al. The role of the late embryogenesis-abundant (LEA) protein family in development and the abiotic stress response: a comprehensive expression analysis of potato (*Solanum Tuberosum*). Genes (Basel). 2019;10(2):148. https://doi.org/10.3390/genes10020148 PMID: 30781418
2. Dure L, Greenway SC, Galau GA. Developmental biochemistry of cottonseed embryogenesis and germination: changing messenger ribonucleic acid populations as shown by in vitro and in vivo protein synthesis. Biochemistry. 1981;20(14):4162-4168. https://doi.org/10.1021/bi00517a033 PMID: 7284317
3. Close TJ, Lammers PJ. An osmotic stress protein of cyanobacteria is immunologically related to plant dehydrins. Plant Physiol. 1993;101(3):773-779. https://doi.org/10.1104/pp.101.3.773 PMID: 8310057
4. Hundertmark M, Hincha DK. LEA (late embryogenesis abundant) proteins and their encoding genes in *Arabidopsis thaliana*. BMC Genomics. 2008;9:118. https://doi.org/10.1186/1471-2164-9-118 PMID: 18318901
5. Wang XS, Zhu HB, Jin GL, Liu HL, Wu WR, Zhu J. Genome-scale identification and analysis of *LEA* genes in rice (*Oryza sativa L.*). Plant Sci. 2007;172(2):414-420. https://doi.org/10.1016/j.plantsci.2006.10.004
6. Zan T, Li L, Li J, Zhang L, Li X. Genome-wide identification and characterization of late embryogenesis abundant protein-encoding gene family in wheat:evolution and expression

profiles during development and stress. Gene. 2020;736:144422. https://doi.org/10.1016/j.gene.2020.144422 PMID: 32007584

7. Amara I, Odena A, Oliveira E, Moreno A, Masmoudi K, Pages M, et al. Insights into maize LEA proteins: from proteomics to functional approaches. Plant Cell Physiol. 2012;53(2):312-329. https://doi.org/10.1093/pcp/pcr183 PMID: 22199372
8. Tunnacliffe A, Lapinski J, McGee B. A putative LEA protein, but no trehalose, is present in anhydrobiotic bdelloid rotifers. Hydrobiologia. 2005;546(1):315-321. https://doi.org/10.1007/s10750-005-4239-6
9. Hand SC, Menze MA, Toner M, Boswell L, Moore D. LEA proteins during water stress: not just for plants anymore. Annu Rev Physiol. 2011;73:115-134. https://doi.org/10.1146/annurev-physiol-012110-142203 PMID: 21034219
10. Hatanaka R, Gusev O, Cornette R, Shimura S, Kikuta S, Okada J, et al. Diversity of the expression profiles of late embryogenesis abundant (LEA) protein encoding genes in the anhydrobiotic midge *Polypedilum vanderplanki*. Planta. 2015;242(2):451-459. https://doi.org/10.1007/s00425-015-2284-6 PMID: 25822798
11. Hunault G, Jaspard E. LEAPdb: a database for the late embryogenesis abundant proteins. BMC Genomics. 2010;11:221. https://doi.org/10.1186/1471-2164-11-221 PMID: 20359361
12. Gonzalez RM, Iusem ND. Twenty years of research on *Asr* (ABA-stress-ripening) genes and proteins. Planta. 2014;239(5):941-949. https://doi.org/10.1007/s00425-014-2039-9 PMID: 24531839
13. Wetzler DE, Fuchs Wightman F, Bucci HA, Rinaldi J, Caramelo JJ, Iusem ND, et al. Conformational plasticity of the intrinsically disordered protein ASR1 modulates its function as a drought stress-responsive gene. PLoS One. 2018;13(8):e0202808. https://doi.org/10.1371/journal.pone.0202808 PMID: 30138481
14. Tunnacliffe A, Wise MJ. The continuing conundrum of the LEA proteins. Naturwissenschaften. 2007;94(10):791-812. https://doi.org/10.1007/s00114-007-0254-y PMID: 17479232
15. Battaglia M, Olvera-Carrillo Y, Garciarrubio A, Campos F, Covarrubias AA. The enigmatic LEA proteins and other hydrophilins. Plant Physiol. 2008;148(1):6-24. https://doi.org/10.1104/pp.108.120725 PMID: 18772351
16. Shao HB, Liang ZS, Shao MA. LEA proteins in higher plants: structure, function, gene

expression and regulation. Colloids Surf B Biointerfaces. 2005;45(3-4):131-135. https://doi.org/10.1016/j.colsurfb.2005.07.017 PMID: 16199145

17. Espelund M, Larssen SS, Hughes DW, Galau GA, Larsen F, Jakobsen KS. Late embryogenesis-abundant genes encoding proteins with different numbers of hydrophilic repeats are regulated differentially by abscisic acid and osmotic stress. Plant J. 1992;2(2):241-252. https://doi.org/10.1046/j.1365-313X.1992.t01-46-00999.x PMID: 1302052
18. Dure L 3rd. A repeating 11-mer amino acid motif and plant desiccation. Plant J. 1993;3(3):363-369. https://doi.org/10.1046/j.1365-313x.1993.t01-19-00999.x PMID: 8220448
19. Close TJ. Dehydrins: Emergence of a biochemical role of a family of plant dehydration proteins. Physiol Plant. 1996;97(4):795-803. https://doi.org/10.1034/j.1399-3054.1996.970422.x
20. Candat A, Paszkiewicz G, Neveu M, Gautier R, Logan DC, Avelange-Macherel MH, et al. The ubiquitous distribution of late embryogenesis abundant proteins across cell compartments in *Arabidopsis* offers tailored protection against abiotic stress. Plant Cell. 2014;26(7):3148-3166. https://doi.org/10.1105/tpc.114.127316 PMID: 25005920
21. Xu D, Duan X, Wang B, Hong B, Ho T, Wu R. Expression of a late embryogenesis abundant protein gene, *HVA1*, from barley confers tolerance to water deficit and salt stress in transgenic rice. Plant Physiol. 1996;110(1):249-257. https://doi.org/10.1104/pp.110.1.249 PMID: 12226181
22. Sivamani E, Bahieldin A, Wraith JM, Al-Niemi T, Dyer WE, Ho THD, et al. Improved biomass productivity and water use efficiency under water deficit conditions in transgenic wheat constitutively expressing the barley *HVA1* gene. Plant Sci. 2000;155(1):1-9. https://doi.org/10.1016/s0168-9452(99)00247-2 PMID: 10773334
23. Hanin M, Brini F, Ebel C, Toda Y, Takeda S, Masmoudi K. Plant dehydrins and stress tolerance. Plant Signal Behav. 2011;6(10):1503-1509. https://doi.org/10.4161/psb.6.10.17088 PMID: 21897131
24. Bauer E, Schmutzer T, Barilar I, Mascher M, Gundlach H, Martis MM, et al. Towards a whole-genome sequence for rye (*Secale cereale* L.). Plant J. 2017;89(5):853-869. https://doi.org/10.1111/tpj.13436 PMID: 27888547
25. Crespo-Herrera LA, Garkava-Gustavsson L, Åhman I. A systematic review of rye (*Secale cereale* L.) as a source of resistance to pathogens and pests in wheat (*Triticum aestivum* L.). Hereditas. 2017;154:14. https://doi.org/10.1186/s41065-017-0033-5 PMID: 28559761

26. Kim W, Johnson JW, Baenziger PS, Lukaszewski AJ, Gaines CS. Agronomic effect of wheat-rye translocation carrying rye chromatin (1R) from different sources. Crop Sci. 2004;44(4):1254-1258. https://doi.org/10.2135/cropsci2004.1254
27. Sharma S, Bhat PR, Ehdaie B, Close TJ, Lukaszewski AJ, Waines JG. Integrated genetic map and genetic analysis of a region associated with root traits on the short arm of rye chromosome 1 in bread wheat. Theor Appl Genet. 2009;119(5):783-793. https://doi.org/10.1007/s00122-009-1088-0 PMID: 19544051
28. NDong C, Danyluk J, Wilson KE, Pocock T, Huner NP, Sarhan F. Cold-regulated cereal chloroplast late embryogenesis abundant-like proteins. Molecular characterization and functional analyses. Plant Physiol. 2002;129(3):1368-1381. https://doi.org/10.1104/pp.001925 PMID: 12114590
29. Katoh K, Standley DM. MAFFT multiple sequence alignment software version 7: improvements in performance and usability. Mol Biol Evol. 2013;30(4):772-780. https://doi.org/10.1093/molbev/mst010 PMID: 23329690
30. Kumar S, Stecher G, Li M, Knyaz C, Tamura K. MEGA X: molecular evolutionary genetics analysis across computing platforms. Mol Biol Evol. 2018;35(6):1547-1549. https://doi.org/10.1093/molbev/msy096 PMID: 29722887
31. Voorrips RE. MapChart: software for the graphical presentation of linkage maps and QTLs. J Hered. 2002;93(1):77-78. https://doi.org/10.1093/jhered/93.1.77 PMID: 12011185
32. Wang Y, Tang H, Debarry JD, Tan X, Li J, Wang X, et al. MCScanX: a toolkit for detection and evolutionary analysis of gene synteny and collinearity. Nucleic Acids Res. 2012;40(7):e49. https://doi.org/10.1093/nar/gkr1293 PMID: 22217600
33. Liao Y, Smyth GK, Shi W. featureCounts: an efficient general purpose program for assigning sequence reads to genomic features. Bioinformatics. 2014;30(7):923-930. https://doi.org/10.1093/bioinformatics/btt656 PMID: 24227677
34. Chen C, Chen H, Zhang Y, Thomas HR, Frank MH, He Y, et al. TBtools - an integrative toolkit developed for interactive analyses of big biological data. Mol Plant. 2020;13(8):1194-1202. https://doi.org/10.1016/j.molp.2020.06.009 PMID: 32585190
35. Schmittgen TD, Livak KJ. Analyzing real-time PCR data by the comparative C_T method. Nat Protoc. 2008;3(6):1101-1108. https://doi.org/10.1038/nprot.2008.73 PMID: 18546601

36. Lan T, Gao J, Zeng QY. Genome-wide analysis of the LEA (late embryogenesis abundant) protein gene family in *Populus trichocarpa*. Tree Genet Genom. 2013;9(1):253-264. https://doi.org/10.1007/s11295-012-0551-2
37. Du D, Zhang Q, Cheng T, Pan H, Yang W, Sun L. Genome-wide identification and analysis of late embryogenesis abundant (LEA) genes in *Prunus mume*. Mol Biol Rep. 2013;40(2):1937-1946. https://doi.org/10.1007/s11033-012-2250-3 PMID: 23086279
38. Fuxreiter M, Simon I, Friedrich P, Tompa P. Preformed structural elements feature in partner recognition by intrinsically unstructured proteins. J Mol Biol. 2004;338(5):1015-1026. https://doi.org/10.1016/j.jmb.2004.03.017 PMID: 15111064
39. Patil A, Nakamura H. Disordered domains and high surface charge confer hubs with the ability to interact with multiple proteins in interaction networks. FEBS Lett. 2006;580(8):2041-2045. https://doi.org/10.1016/j.febslet.2006.03.003 PMID: 16542654
40. Holub EB. The arms race is ancient history in *Arabidopsis*, the wildflower. Nat Rev Genet. 2001;2(7):516-527. https://doi.org/10.1038/35080508 PMID: 11433358
41. Dvorak J, Fowler DB. Cold hardiness potential of Triticale and Teraploid Rye. Crop Sci. 1978;18(3):477-478. https://doi.org/10.2135/cropsci1978.0011183x001800030030x
42. Liang Y, Xiong Z, Zheng J, Xu D, Zhu Z, Xiang J, et al. Genome-wide identification, structural analysis and new insights into late embryogenesis abundant (LEA) gene family formation pattern in *Brassica napus*. Sci Rep. 2016;6:24265. https://doi.org/10.1038/srep24265 PMID: 27072743
43. Hu R, Qi G, Kong Y, Kong D, Gao Q, Zhou G. Comprehensive analysis of NAC domain transcription factor gene family in *Populus trichocarpa*. BMC Plant Biol. 2010;10:145. https://doi.org/10.1186/1471-2229-10-145 PMID: 20630103
44. Bies-Ethève N, Gaubier-Comella P, Debures A, Lasserre E, Jobet E, Raynal M, et al. Inventory, evolution and expression profiling diversity of the LEA (late embryogenesis abundant) protein gene family in *Arabidopsis thaliana*. Plant Mol Biol. 2008;67:107-124. https://doi.org/10.1007/s11103-008-9304-x PMID: 18265943
45. Cao J, Li X. Identification and phylogenetic analysis of late embryogenesis abundant proteins family in tomato (*Solanum lycopersicum*). Planta. 2015;241(3):757-772. https://doi.org/10.1007/s00425-014-2215-y PMID: 25491641
46. Bartos J, Paux E, Kofler R, Havrankova M, Kopecky D, Suchankova P, et al. A first survey of the

rye (*Secale cereale*) genome composition through BAC end sequencing of the short arm of chromosome 1R. BMC Plant Biol. 2008;8:95. https://doi.org/10.1186/1471-2229-8-95 PMID: 18803819

47. Moore RC, Purugganan MD. The early stages of duplicate gene evolution. PNAS. 2004;100(26):15682-15687. https://doi.org/10.1073/pnas.2535513100 PMID: 14671323
48. Ling H, Zeng X, Guo S. Functional insights into the late embryogenesis abundant (LEA) protein family from *Dendrobium officinale* (Orchidaceae) using an *Escherichia coli* system. Sci Rep. 2016;6(1):39693. https://doi.org/10.1038/srep39693 PMID: 28004781
49. Liu D, Sun J, Zhu D, Lyu G, Zhang C, Liu J, et al. Genome-wide identification and expression profiles of late embryogenesis-abundant (LEA) genes during grain maturation in wheat (*Triticum aestivum* L.). Genes (Basel). 2019;10(9):696. https://doi.org/10.3390/genes10090696 PMID: 31510067
50. Xiao B, Huang Y, Tang N, Xiong L. Over-expression of a *LEA* gene in rice improves drought resistance under the field conditions. Theor Appl Genet. 2007;115(1):35-46. https://doi.org/10.1007/s00122-007-0538-9 PMID: 17426956
51. Franklin KA, Quail PH. Phytochrome functions in *Arabidopsis* development. J Exp Bot. 2010;61(1):11-24. https://doi.org/10.1093/jxb/erp304 PMID: 19815685
52. He S, Tan L, Hu Z, Chen G, Wang G, Hu T. Molecular characterization and functional analysis by heterologous expression in *E. coli* under diverse abiotic stresses for *OsLEA5*, the atypical hydrophobic LEA protein from *Oryza sativa* L. Mol Genet Genomics. 2012;287(1):39-54. https://doi.org/10.1007/s00438-011-0660-x PMID: 22127413
53. Huwaidi A, Pathak N, Syahir A, Ikeno S. *Escherichia coli* tolerance of ultraviolet radiation by in vivo expression of a short peptide designed from late embryogenesis abundant protein. Biochem Biophys Res Commun. 2018;503(2):910-914. https://doi.org/10.1016/j.bbrc.2018.06.095 PMID: 29928878
54. Hübner M, Wilde P, Schmiedchen B, Dopierala P, Gowda M, Reif JC, et al. Hybrid rye performance under natural drought stress in Europe. Theor Appl Genet. 2013;126(2):475-482. https://doi.org/10.1007/s00122-012-1994-4 PMID: 23090142
55. Sachs MM, Ho T-HD. Alteration of gene expression during environmental stress in plants. Ann Rev Plant Physiol. 1986;37(1):363-376. https://doi.org/10.1146/annurev.pp.37.060186.002051

56. Mitchell PJ, Tjian R. Transcriptional regulation in mammalian cells by sequence-specific DNA binding proteins. Science. 1989;245(4916):371-378. https://doi.org/10.1126/science.2667136 PMID: 2667136

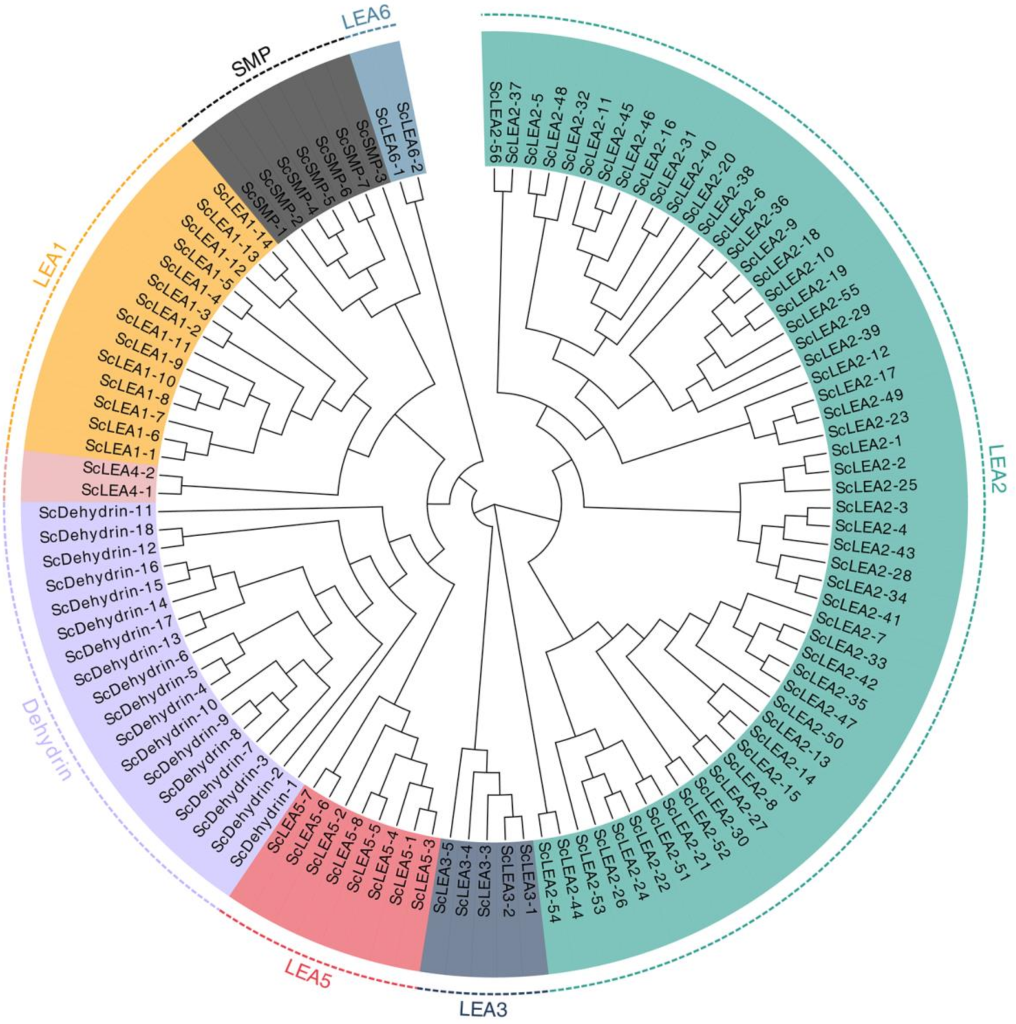

Figure 1. Phylogenetic analysis of ScLEA proteins. Multiple sequence alignment of LEA proteins was performed using MAFFT. The phylogenetic tree was constructed using MEGA X software.

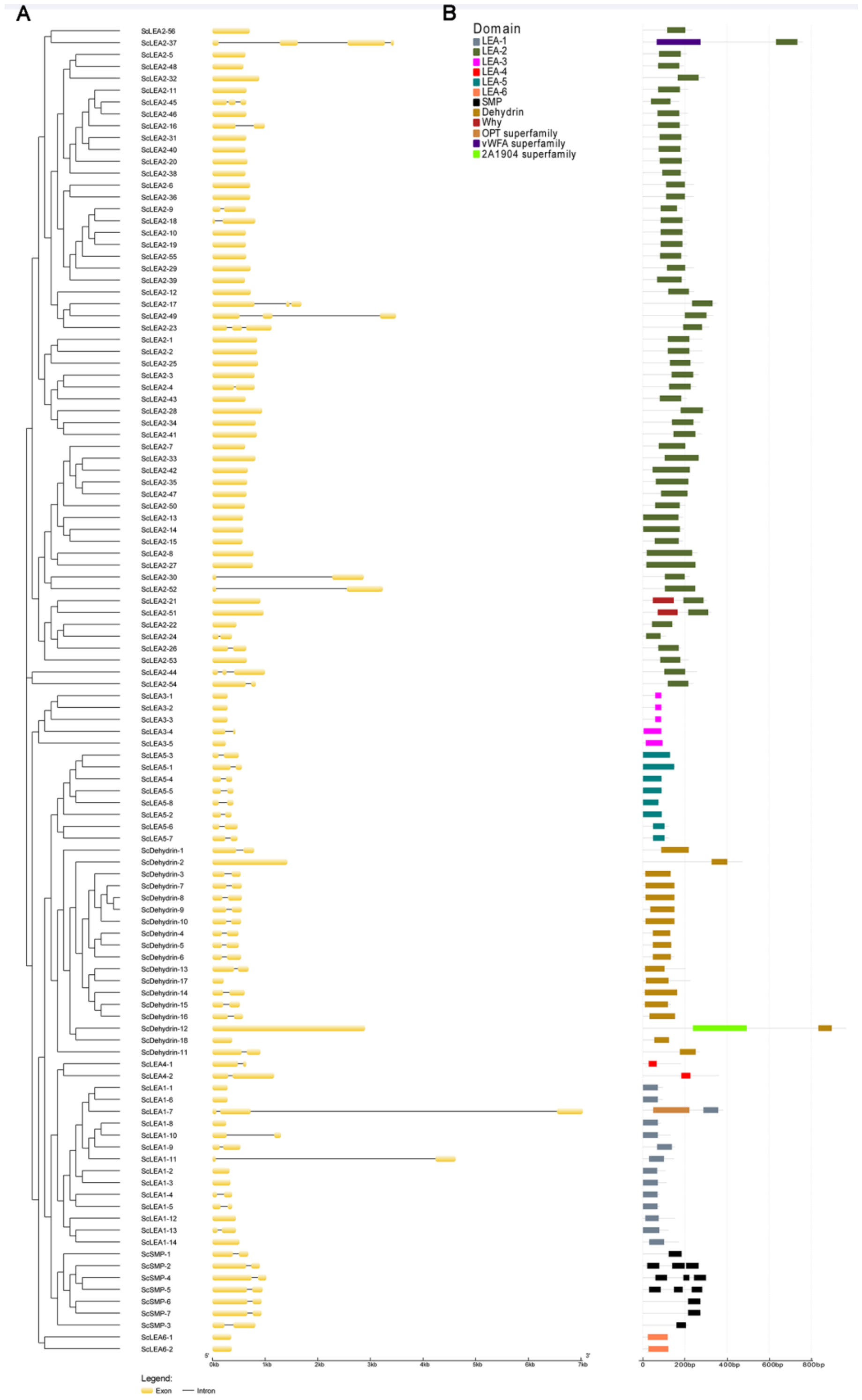

Figure 2. Schematic representation of exon-intron structures of *ScLEA* genes and conserved domains of ScLEA proteins. (**a**) The exon-intron structures were examined using the GSDS online tool. The yellow boxes and black lines represent exons and introns, respectively. **(b)** The ScLEA protein domains were predicted using the MEME online tool.

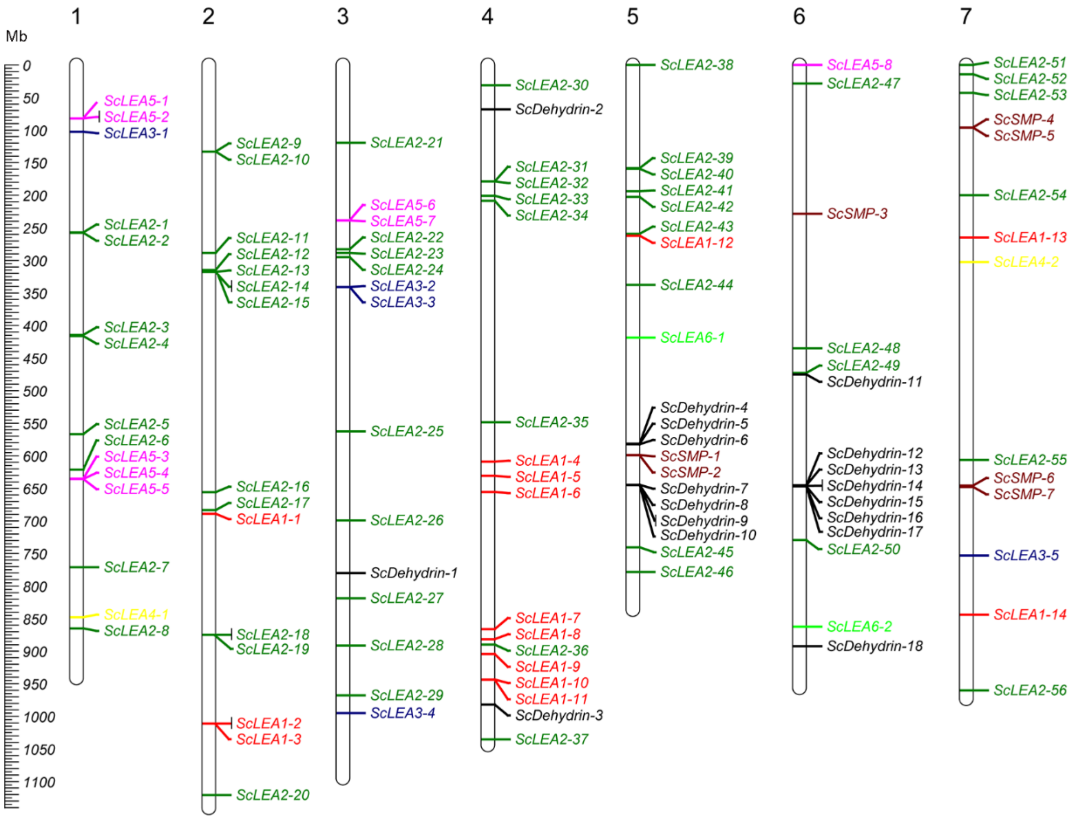

Figure 3. Distribution of *ScLEA* genes on rye chromosomes visualized using Mapchart software.

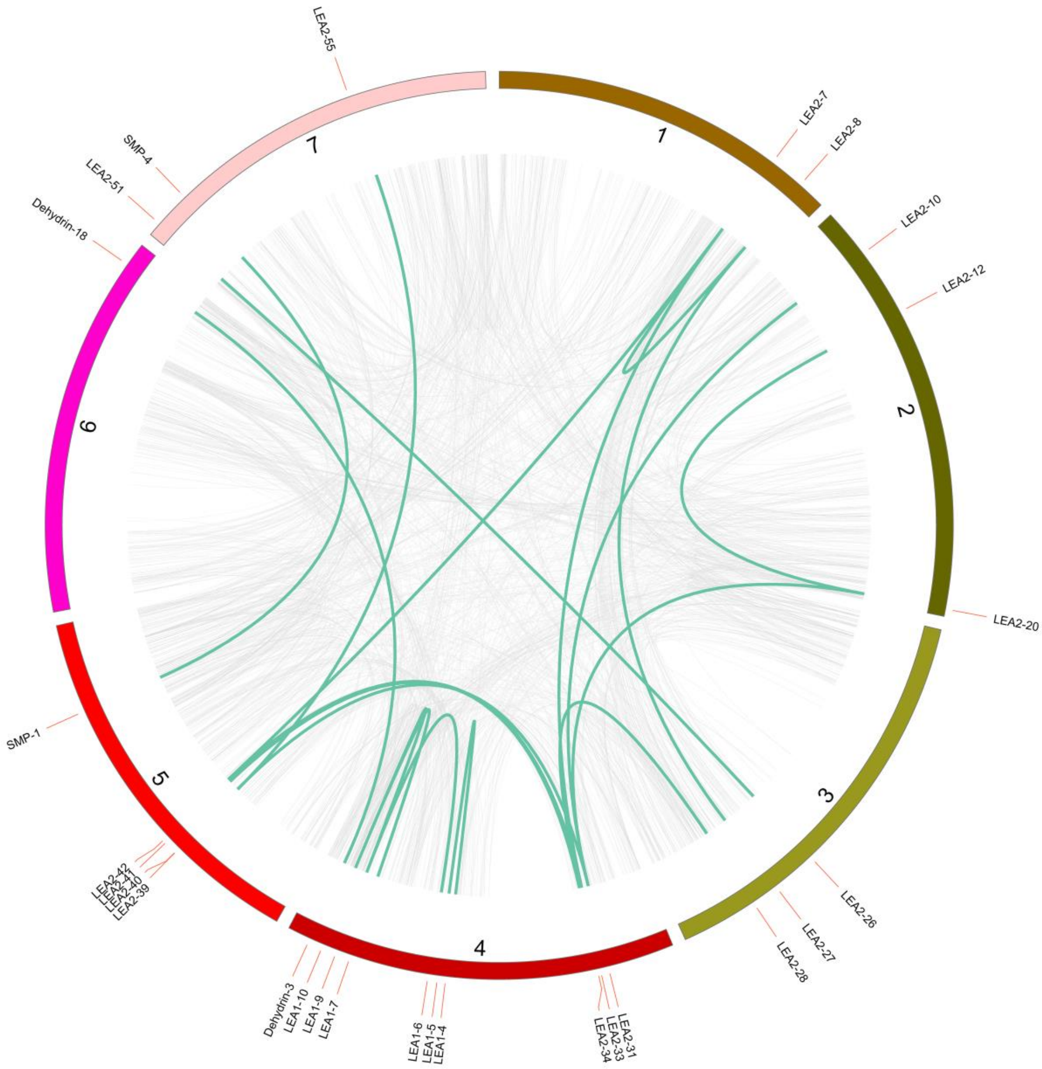

Figure 4. Schematic representation of the chromosomal distribution and interchromosomal relationships of *ScLEA* genes derived from segmental duplication. The gray lines indicate all syntenic gene pairs in the rye genome, and the dark turquoise lines indicate segmental duplicated *ScLEA* gene pairs. The number of each chromosome is indicated.

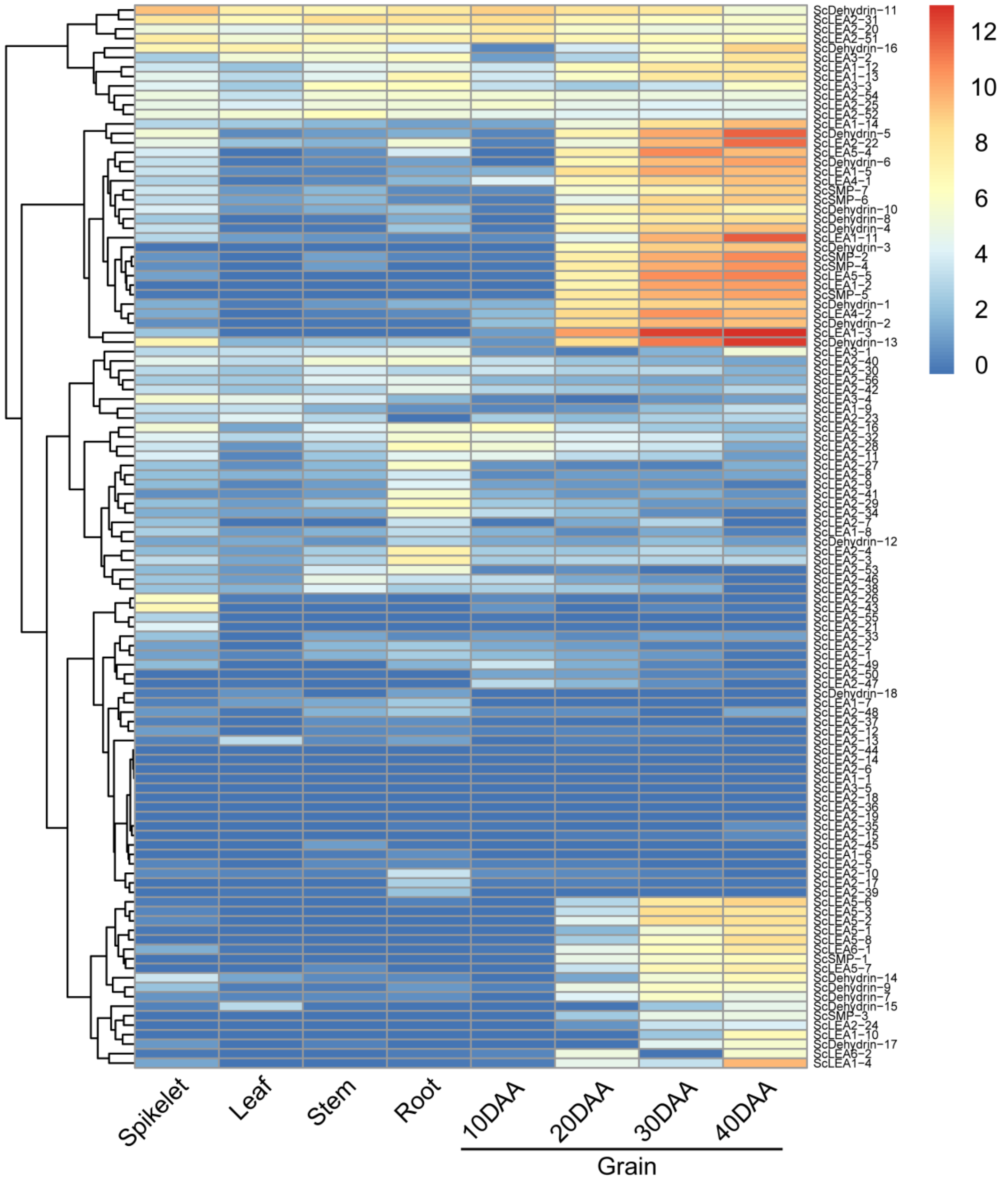

Figure 5. RNA-seq expression profiles of 112 *ScLEA* genes in different tissues (spikelet, leaf, stem, root, and grain) of rye (Weining). The heatmap was constructed using TBtools. The color scale on the right represents relative expression levels: red represents high level and blue represents low level.

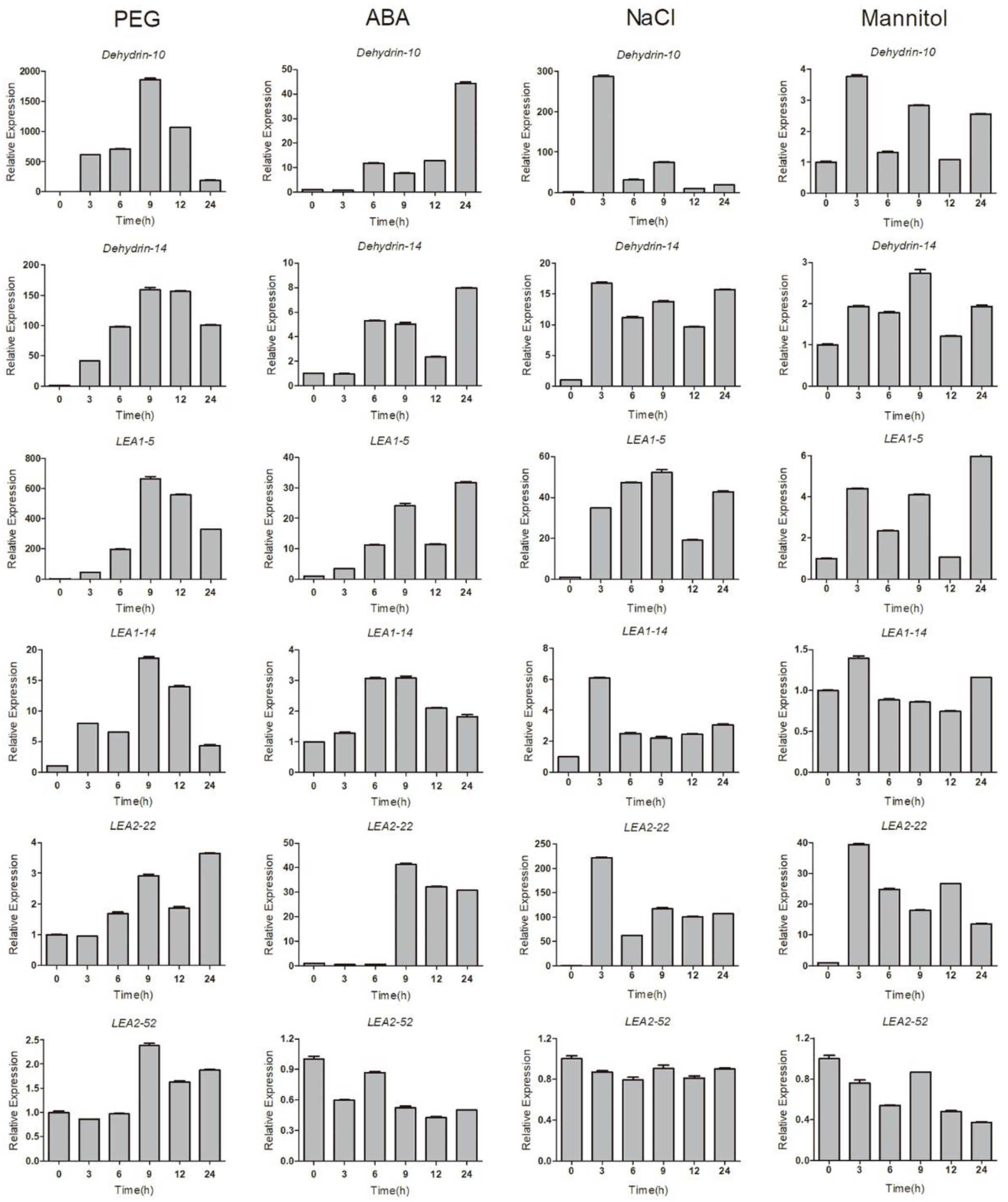

Figure 6. The relative expression levels of six *ScLEA* genes (*ScDehydrin-10, ScDehydrin-14, ScLEA1-5, ScLEA1-14, ScLEA2-22,* and *ScLEA2-52*) under different abiotic stresses were analyzed by qRT-PCR. Ten-day-old seedling leaves were sampled after 0 h, 3 h, 6 h, 9 h, 12 h, and 24 h under 20% PEG6000, 100 μM ABA, 200 mM NaCl, or 100 mM mannitol. The values represent mean ± SEM of three replicates.

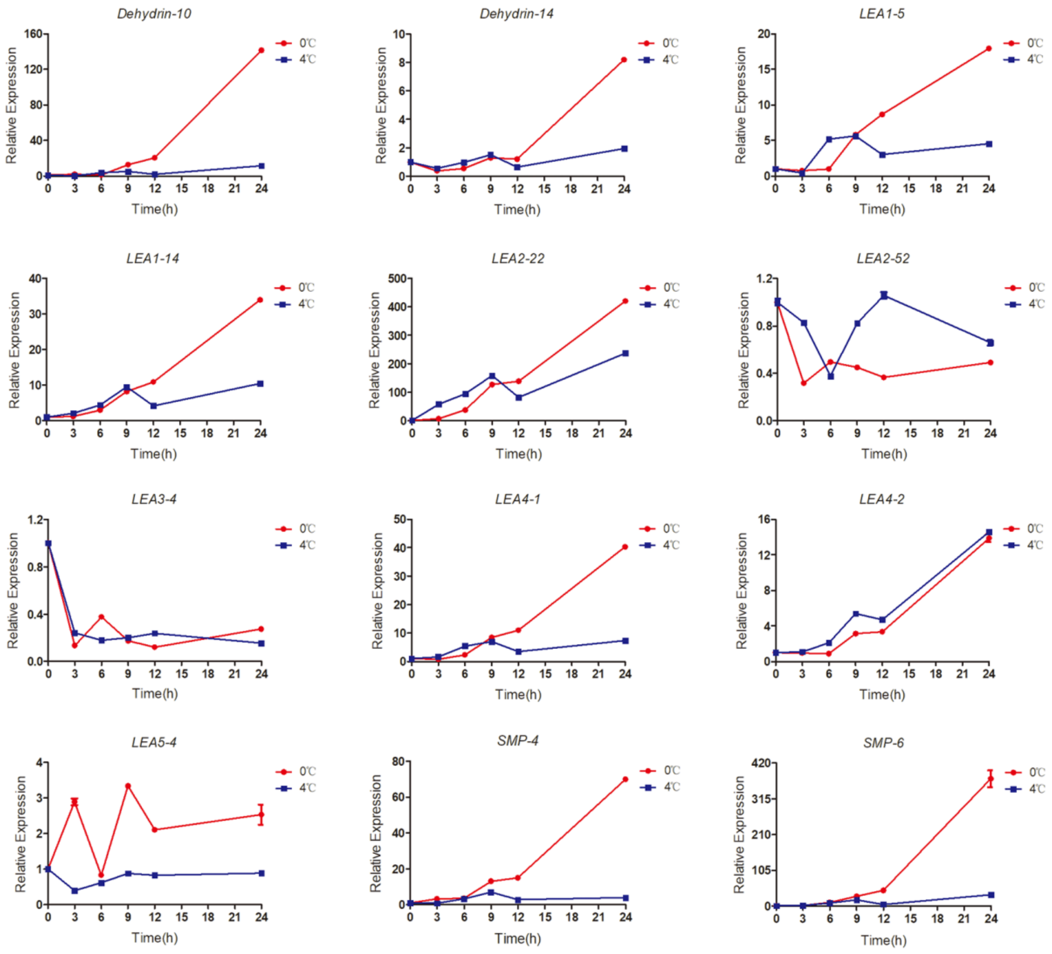

Figure 7. The relative expression levels of 12 *ScLEA* genes (*ScDehydrin-10, ScDehydrin-14, ScLEA1-5, ScLEA1-14, ScLEA2-22, ScLEA2-52, ScLEA3-4, ScLEA4-1, ScLEA4-2, ScLEA5-4, ScSMP-4*, and *ScSMP-6*) under cold stress were analyzed by qRT-PCR. Ten-day-old seedling leaves were sampled after 0 h, 3 h, 6 h, 9 h, 12 h, and 24 h at 0°C or 4℃. The values represent mean ± SEM of three replicates.

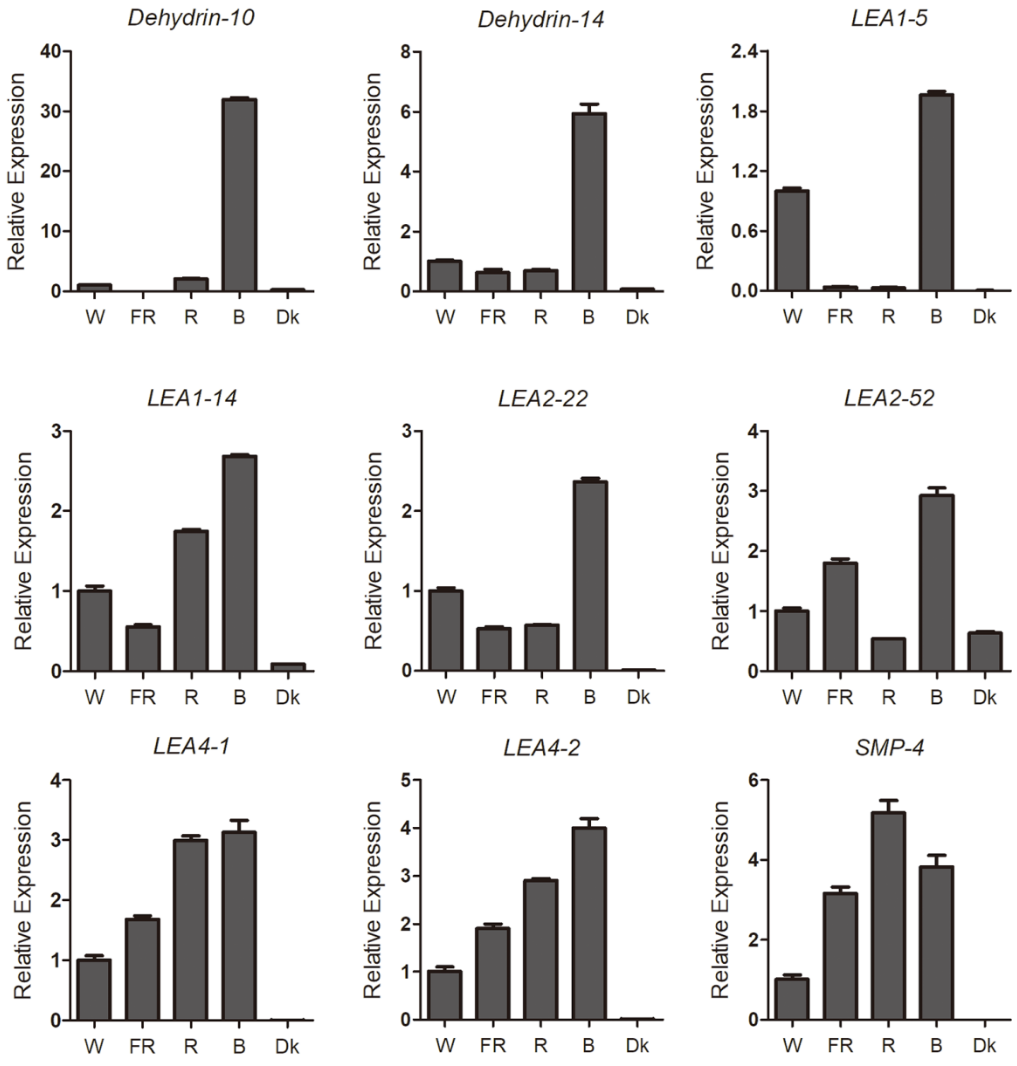

Figure 8. The relative expression levels of nine *ScLEA* genes (*ScDehydrin-10, ScDehydrin-14, ScLEA1-5, ScLEA1-14, ScLEA2-22, ScLEA2-52, ScLEA4-1, ScLEA4-2*, and *ScSMP-4*) under different light conditions were analyzed by qRT-PCR. Seedlings were grown in the dark for seven days and subsequently transferred to FR light (5 $\mu mol \cdot m^{-2} \cdot s^{-1}$), R light (17.56 $\mu mol \cdot m^{-2} \cdot s^{-1}$), B light (13 $\mu mol \cdot m^{-2} \cdot s^{-1}$), or W light (85 $\mu mol \cdot m^{-2} \cdot s^{-1}$) for 4 h. The values represent mean ± SEM of three replicates.

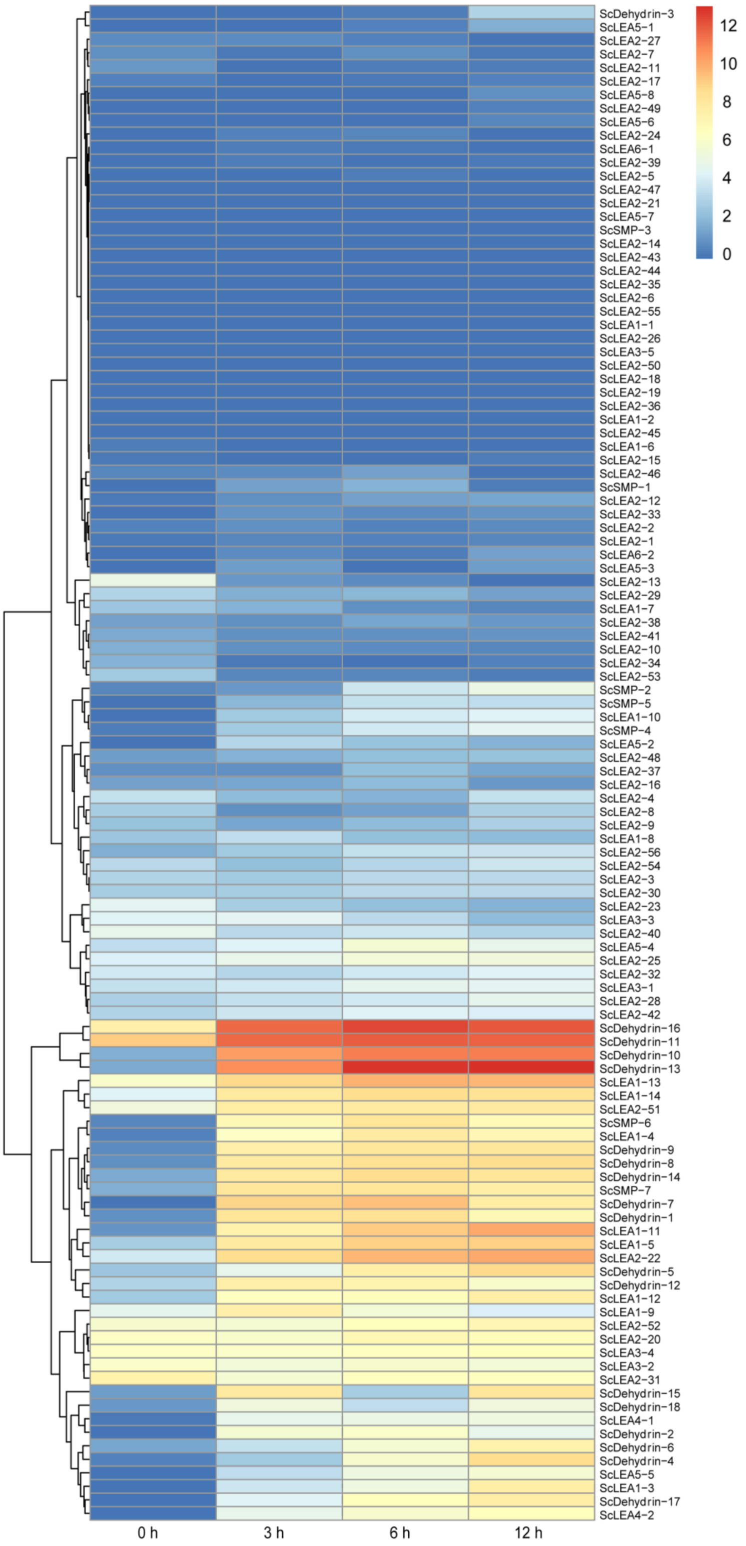

Figure S1. RNA-seq expression profiles of 112 *ScLEA* genes after drought treatment for 0 h, 3 h, 6 h, and 12 h. The heatmap was constructed using TBtools. The color scale on the right represents relative expression levels: red represents high level and blue represents low level.

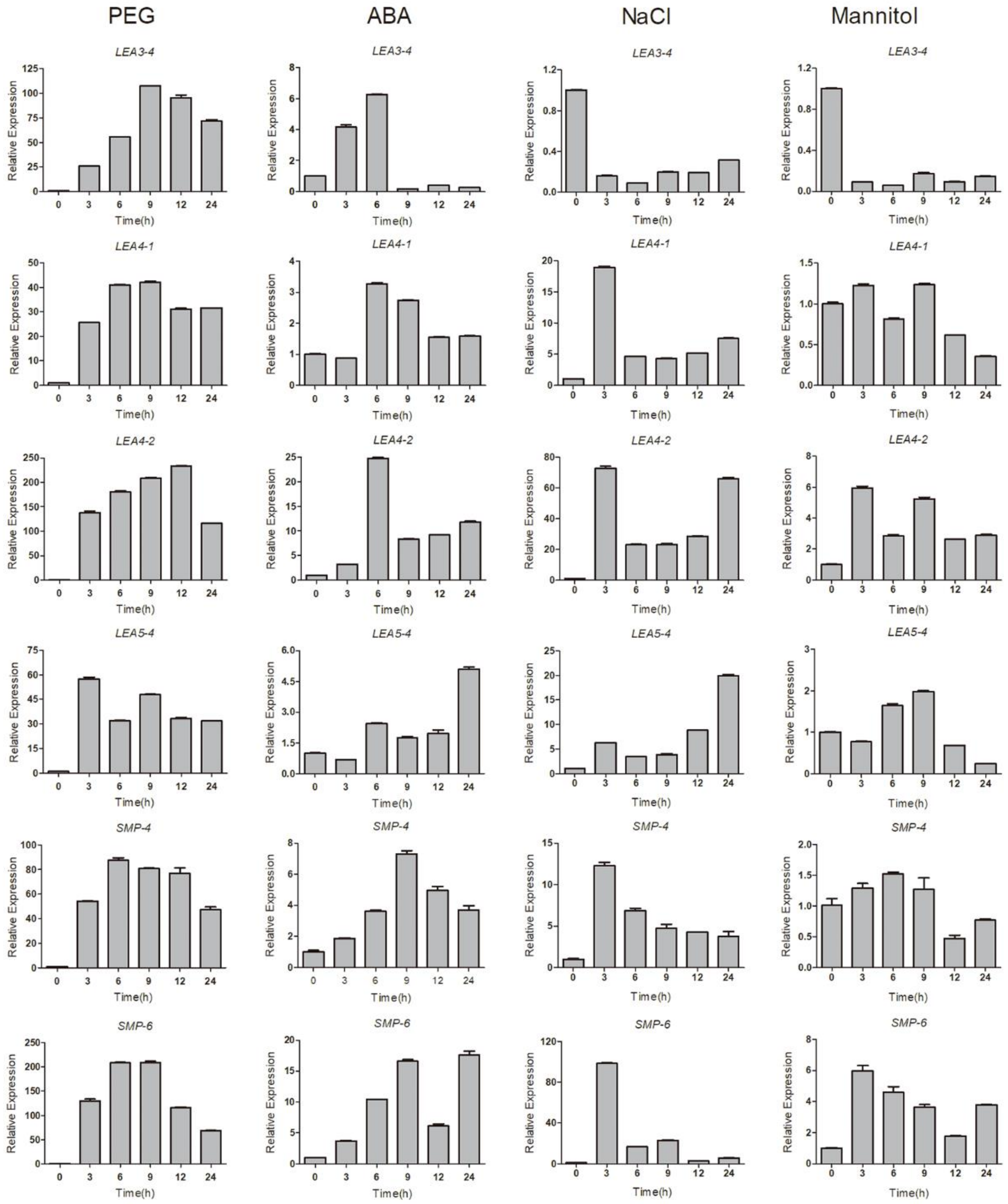

Figure S2. The relative expression levels of six *ScLEA* genes (*ScLEA3-4*, *ScLEA4-1*, *ScLEA4-2*, *ScLEA5-4*, *ScSMP-4*, and *ScSMP-6*) under different abiotic stresses were analyzed by qRT-PCR. Ten-day-old seedling leaves were sampled after 0 h, 3 h, 6 h, 9 h, 12 h, and 24 h under 20% PEG6000, 100 μM ABA, 200 mM NaCl, or 100 mM mannitol. The values represent mean ± SEM of three replicates.

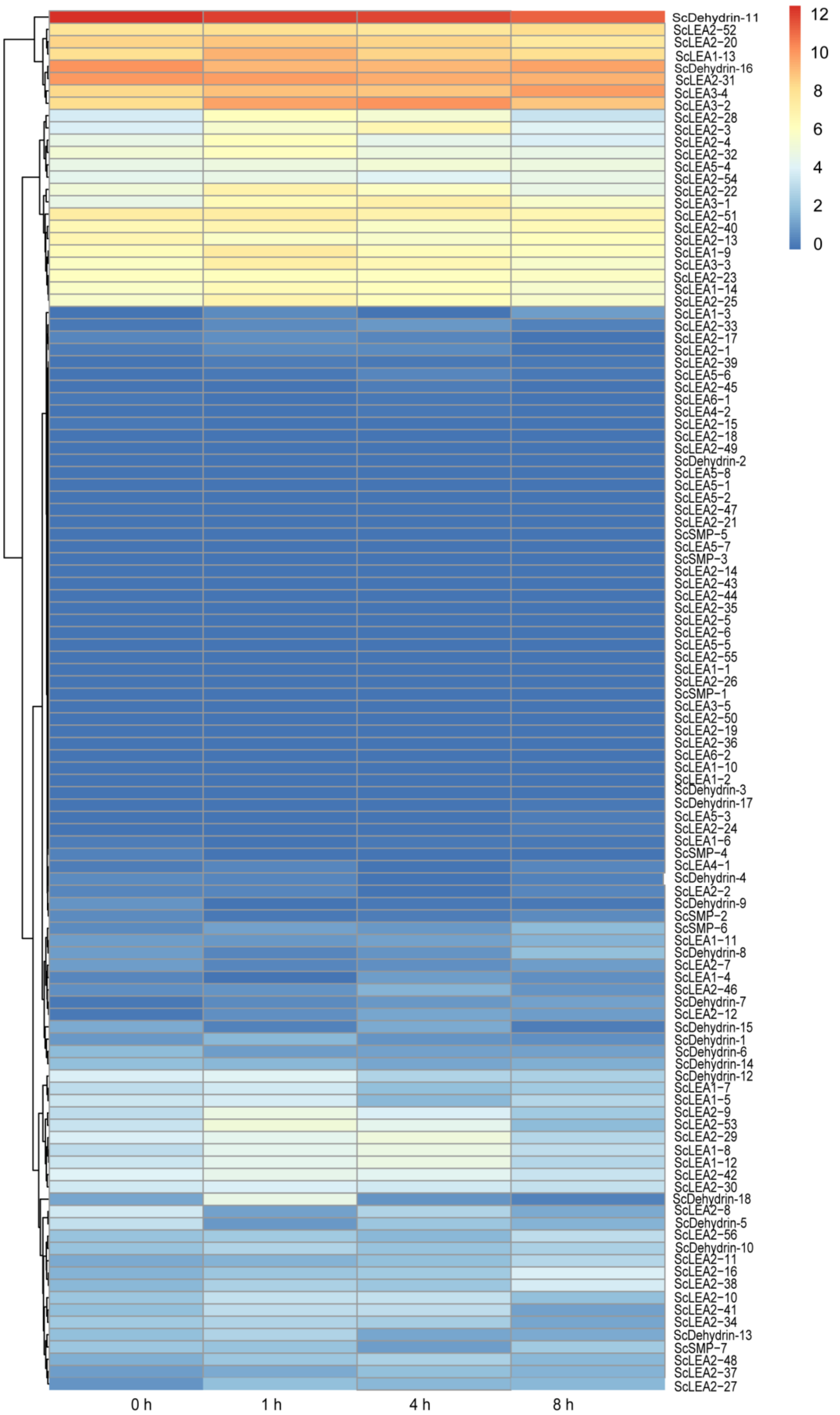

Figure S3. RNA-seq expression profiles of 112 *ScLEA* genes after incubation at -10℃ for 0 h, 1 h, 4 h, and 8 h. The heatmap was constructed using TBtools. The color scale on the right represents relative expression levels: red represents high level and blue represents low level.

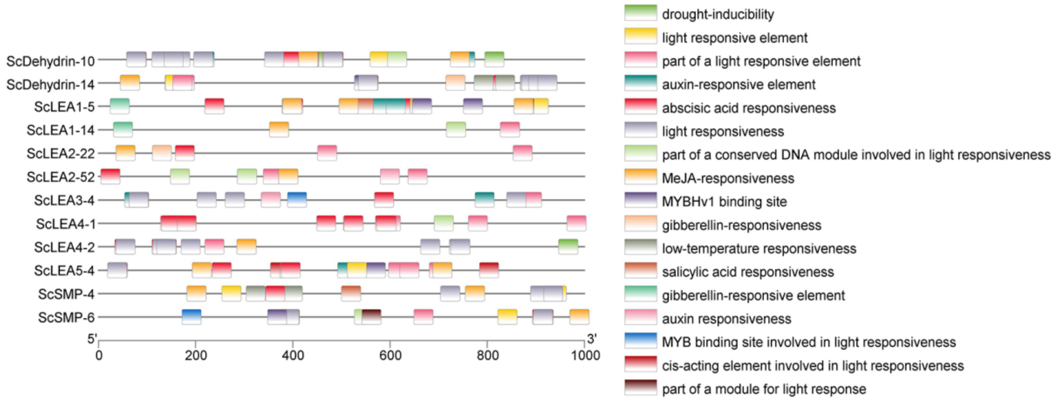

Figure S4. Predicted *cis*-elements in the promoters of 12 *ScLEA* genes (*ScDehydrin-10*, *ScDehydrin-14*, *ScLEA1-5*, *ScLEA1-14*, *ScLEA2-22*, *ScLEA2-52*, *ScLEA3-4*, *ScLEA4-1*, *ScLEA4-2*, *ScLEA5-4*, *ScSMP-4*, and *ScSMP-6*).

Table S1. List of qRT-PCR primers for the 12 *ScLEA* genes in this study.

Gene	Forward primer（5'–3'）	Reverse primer（5'–3'）
ScActin	CGTGTTGGATTCTGGTGATG	AGCCACATATGCGAGCTTCT
ScDehydrin-10	AGCTCTAGCTCGTCTTCCG	TGTGCTGCTGGTTGTCCT
ScDehydrin-14	TAGTAGCTCCAGCTCGTC	TTCTCCTTGATCTTATCCTTGAT
ScLEA1-5	TCAAAGCGAAGGTCCAGG	ATGCGGTCCTCCTTGTTT
ScLEA1-14	GATAGCATCCGCTAAAATCTG	GGCCTGCATGTTGTCTAG
ScLEA2-22	ACATACCAGTGAAGGTGC	GTAGTCGAGGTCCCAGTC
ScLEA2-52	GTTCCATTTCAACATCCC	AATCCTGACTTATGATACTCT
ScLEA3-4	CAGCCTCCTCGCACAAA	ACGGCGGTCTTCTCCTC
ScLEA4-1	GAACACGCTGGGCATGAG	TGATCCCTGGTGATCTTCTC
ScLEA4-2	GACTACACCGTCGAGAAG	TTGTTCTTGGTAGCCTCA
ScLEA5-4	ATGGCCTCCGGTCAGC	CCCTTCGGCGAGGTTCT
ScSMP-4	CTGTACTACTCACCTAGCC	CTCATCTTGGCGAGAAGG
ScSMP-6	GGCATCACCGTCACCCAGAC	GCTCGTGTCCTCCTCCTCCA

Table S2. List of the 112 *ScLEA* genes identified in this study.

Gene Name	Gene ID	Amino Acid Number	Molecular Weight (Da)	pI	Aliphatic index	GRAVY
ScDehydrin-1	ScWN3R01G346600	218	22423.55	6.25	49.31	-0.811
ScDehydrin-2	ScWN4R01G072800	473	45913.81	8.88	42.52	-0.618
ScDehydrin-3	ScWN4R01G570900	132	13466.8	9.56	41.59	-1.041
ScDehydrin-4	ScWN5R01G416400	132	13252.44	8.81	37.12	-1.034
ScDehydrin-5	ScWN5R01G416500	137	13786.03	8.81	40	-0.978
ScDehydrin-6	ScWN5R01G417300	149	15098.56	6.71	49.19	-0.766
ScDehydrin-7	ScWN5R01G479300	150	15133.48	9.22	35.27	-1.026
ScDehydrin-8	ScWN5R01G479400	150	15171.57	9.16	35.93	-1.017
ScDehydrin-9	ScWN5R01G479800	150	15207.62	9.19	33.33	-1.062
ScDehydrin-10	ScWN5R01G479900	150	15112.52	9.4	33.33	-1.044
ScDehydrin-11	ScWN6R01G204000	270	28839.07	5.23	63.26	-0.942
ScDehydrin-12	ScWN6R01G318400	965	99640.75	5.51	29.52	-1.255
ScDehydrin-13	ScWN6R01G318700	204	20313.83	9.07	26.86	-1.039
ScDehydrin-14	ScWN6R01G319200	163	16428.76	9.19	34.85	-1.045
ScDehydrin-15	ScWN6R01G319400	134	13332.25	6.75	34.33	-1.004
ScDehydrin-16	ScWN6R01G319500	153	15520.78	8.86	33.27	-1.098
ScDehydrin-17	ScWN6R01G319600	226	23330.67	9.58	47.12	-0.783
ScDehydrin-18	ScWN6R01G557000	124	12724.73	6.34	37.74	-1.157
ScLEA1-1	ScWN2R01G334900	95	10186.65	10.24	65.05	-0.826
ScLEA1-2	ScWN2R01G550900	107	11347.61	9.16	43.18	-0.998
ScLEA1-3	ScWN2R01G551000	113	11760.1	9.58	38.41	-1.048
ScLEA1-4	ScWN4R01G263400	80	8600.55	8.96	50.25	-1.188
ScLEA1-5	ScWN4R01G275600	80	8600.55	8.96	50.25	-1.188
ScLEA1-6	ScWN4R01G288100	95	10186.65	10.24	65.05	-0.826
ScLEA1-7	ScWN4R01G457200	381	42198.19	9.55	80.97	-0.385

ScLEA1-8	ScWN4R01G467600	86	9472.7	9.74	58.26	-1.088
ScLEA1-9	ScWN4R01G486600	154	17256.57	10.64	45.26	-1.297
ScLEA1-10	ScWN4R01G530800	133	15106.17	9.86	49.4	-1.092
ScLEA1-11	ScWN4R01G531000	148	15581.87	9.93	61.62	-0.732
ScLEA1-12	ScWN5R01G179500	153	15356.83	8.07	52.68	-0.697
ScLEA1-13	ScWN7R01G229800	122	12920.6	9.37	72.21	-0.435
ScLEA1-14	ScWN7R01G451300	170	16807.66	9.56	57.24	-0.436
ScLEA2-1	ScWN1R01G171300	282	29488.16	9.92	73.44	-0.003
ScLEA2-2	ScWN1R01G171400	282	29488.16	9.92	73.44	-0.003
ScLEA2-3	ScWN1R01G204500	266	28783.43	10.21	95.68	0.017
ScLEA2-4	ScWN1R01G206300	254	27538.08	10.32	97.13	0.015
ScLEA2-5	ScWN1R01G282200	208	22310.79	9.65	102.07	0.262
ScLEA2-6	ScWN1R01G311600	241	26955.15	8.92	91.37	0.037
ScLEA2-7	ScWN1R01G410500	206	21639.30	10.93	104.61	0.303
ScLEA2-8	ScWN1R01G495100	259	26739.67	8.89	95.68	0.224
ScLEA2-9	ScWN2R01G118100	187	19935.63	9.39	96.63	0.491
ScLEA2-10	ScWN2R01G118200	210	22318.15	9.05	96.24	0.393
ScLEA2-11	ScWN2R01G216500	214	22933.59	9.30	94.21	0.170
ScLEA2-12	ScWN2R01G231100	242	26768.59	9.26	79.01	-0.223
ScLEA2-13	ScWN2R01G232500	192	20580.09	8.98	111.41	0.474
ScLEA2-14	ScWN2R01G232600	195	20865.41	9.14	107.69	0.529
ScLEA2-15	ScWN2R01G232700	190	19936.41	10.54	107.42	0.502
ScLEA2-16	ScWN2R01G313600	216	23193.91	8.77	101.06	0.301
ScLEA2-17	ScWN2R01G330800	351	38093.33	11.07	68.12	-0.576
ScLEA2-18	ScWN2R01G451300	222	23777.88	8.83	98.51	0.383
ScLEA2-19	ScWN2R01G451400	210	22378.15	9.05	96.24	0.393
ScLEA2-20	ScWN2R01G656000	221	24361.35	9.01	95.70	0.178
ScLEA2-21	ScWN3R01G125000	305	33669.17	4.69	92.98	-0.335
ScLEA2-22	ScWN3R01G222500	151	16214.62	4.89	94.24	-0.032

ScLEA2-23	ScWN3R01G225000	314	33856.14	9.02	76.97	-0.246
ScLEA2-24	ScWN3R01G228600	111	12079.84	4.23	112.34	0.226
ScLEA2-25	ScWN3R01G279000	288	29844.54	10.15	78.96	0.085
ScLEA2-26	ScWN3R01G305900	181	19394.92	4.45	81.82	-0.125
ScLEA2-27	ScWN3R01G370500	255	27270.63	4.85	88.24	-0.077
ScLEA2-28	ScWN3R01G419400	314	33669.44	9.73	78.31	-0.120
ScLEA2-29	ScWN3R01G464400	241	26377.37	9.74	79.38	-0.166
ScLEA2-30	ScWN4R01G039100	223	24048.03	9.54	116.77	0.262
ScLEA2-31	ScWN4R01G143600	213	23156.67	8.67	103.47	0.272
ScLEA2-32	ScWN4R01G143700	295	30972.13	9.67	74.95	-0.149
ScLEA2-33	ScWN4R01G156400	271	29238.82	10.53	88.15	-0.067
ScLEA2-34	ScWN4R01G161000	273	29124.74	10.19	94.80	0.046
ScLEA2-35	ScWN4R01G228000	219	23463.46	9.74	106.85	0.252
ScLEA2-36	ScWN4R01G475300	241	26955.15	8.92	91.37	0.037
ScLEA2-37	ScWN4R01G625900	759	83942.38	8.50	94.01	-0.129
ScLEA2-38	ScWN5R01G100700	208	21840.82	9.48	94.57	0.030
ScLEA2-39	ScWN5R01G133100	205	22029.42	8.79	94.63	0.190
ScLEA2-40	ScWN5R01G133200	208	22731.02	8.60	100.24	0.141
ScLEA2-41	ScWN5R01G146300	280	30370.01	10.41	81.61	-0.128
ScLEA2-42	ScWN5R01G150500	223	23785.49	9.38	98.37	0.122
ScLEA2-43	ScWN5R01G178000	209	22789.23	6.88	83.44	-0.077
ScLEA2-44	ScWN5R01G227200	257	27924.60	9.34	101.28	0.167
ScLEA2-45	ScWN5R01G574600	172	18082.92	9.46	94.63	0.254
ScLEA2-46	ScWN5R01G618300	214	22593.09	9.40	97.38	0.231
ScLEA2-47	ScWN6R01G075200	215	23324.15	8.94	102.47	0.127
ScLEA2-48	ScWN6R01G183100	195	21542.22	10.61	96.46	0.053
ScLEA2-49	ScWN6R01G202600	335	36099.93	9.86	73.13	-0.236
ScLEA2-50	ScWN6R01G399400	204	21250.67	10.26	114.66	0.496
ScLEA2-51	ScWN7R01G051300	325	35951.80	4.90	90.80	-0.394

ScLEA2-52	ScWN7R01G066100	250	26849.30	9.88	103.84	0.195
ScLEA2-53	ScWN7R01G092200	217	23268.20	9.34	99.03	0.197
ScLEA2-54	ScWN7R01G192900	239	25366.38	8.93	100.79	0.304
ScLEA2-55	ScWN7R01G312400	213	23379.12	8.63	94.79	0.255
ScLEA2-56	ScWN7R01G555200	235	26002.43	10.07	96.68	0.108
ScLEA3-1	ScWN1R01G098100	95	9429.5	10.46	66.53	-0.226
ScLEA3-2	ScWN3R01G245700	95	9429.5	10.46	66.53	-0.226
ScLEA3-3	ScWN3R01G245900	95	9451.51	10.46	66.53	-0.246
ScLEA3-4	ScWN3R01G482900	95	9968.48	9.74	65.05	-0.273
ScLEA3-5	ScWN7R01G388500	97	10547.14	9.65	74.74	-0.137
ScLEA4-1	ScWN1R01G477800	180	18645.24	5.62	41.61	-0.998
ScLEA4-2	ScWN7R01G243600	362	38447.1	6.45	47.27	-1.017
ScLEA5-1	ScWN1R01G081900	153	16911.43	5.45	30.65	-1.551
ScLEA5-2	ScWN1R01G082000	94	9967.75	5.25	37.45	-1.286
ScLEA5-3	ScWN1R01G319500	133	14637.96	5.42	32.33	-1.489
ScLEA5-4	ScWN1R01G319900	93	10003.79	5.56	37.85	-1.403
ScLEA5-5	ScWN1R01G320000	93	9994.84	5.29	37.85	-1.317
ScLEA5-6	ScWN3R01G198900	124	13554.98	4.85	69.35	-1.18
ScLEA5-7	ScWN3R01G199300	124	13643	4.73	67.02	-1.267
ScLEA5-8	ScWN6R01G061600	78	8235.95	4.97	45.13	-1.105
ScLEA6-1	ScWN5R01G284200	118	12485.53	5.66	53.05	-1.014
ScLEA6-2	ScWN6R01G525700	121	13038.07	5.24	52.56	-1.121
ScSMP-1	ScWN5R01G432400	189	19092.07	5.04	73.7	-0.292
ScSMP-2	ScWN5R01G432600	266	27096.24	5.15	77.56	-0.325
ScSMP-3	ScWN6R01G127300	215	22870.59	10.96	65.58	-0.682
ScSMP-4	ScWN7R01G131900	302	30451.8	5.74	74.24	-0.294
ScSMP-5	ScWN7R01G132000	284	28975.85	4.94	69.65	-0.434
ScSMP-6	ScWN7R01G333200	278	28537.13	4.22	66.98	-0.39
ScSMP-7	ScWN7R01G335400	278	28479.09	4.24	67.34	-0.371

Table S3. Tandem duplicated *ScLEA* genes.

Gene	Gene	Gene	Gene	Chromosome location	Chromosomal region (bp)
ScLEA5-1	*ScLEA5-2*			Chr1	4917
ScLEA5-4	*ScLEA5-5*			Chr1	83114
ScLEA2-9	*ScLEA2-10*			Chr2	36748
ScLEA2-18	*ScLEA2-19*			Chr2	36941
ScLEA1-2	*ScLEA1-3*			Chr2	4386
ScLEA3-2	*ScLEA3-3*			Chr3	61746
ScLEA2-31	*ScLEA2-32*			Chr4	11843
ScDehydrin-12	*ScDehydrin-13*			Chr6	87935
ScSMP-1	*ScSMP-2*			Chr5	94940
ScLEA2-13	*ScLEA2-14*	*ScLEA2-15*		Chr2	163748
ScDehydrin-14	*ScDehydrin-15*	*ScDehydrin-16*		Chr6	89392
ScDehydrin-7	*ScDehydrin-8*	*ScDehydrin-9*	*ScDehydrin-10*	Chr5	125733

Table S4. Segmental duplicated *ScLEA* genes.

Gene	Chromosome location	Gene	Chromosome location	E-value
ScLEA2-7	chr1	*ScLEA2-8*	chr1	6.00E-12
ScLEA2-8	chr1	*ScLEA2-27*	chr3	4.00E-82
ScLEA2-7	chr1	*ScLEA2-33*	chr4	9.00E-35
ScLEA2-7	chr1	*ScLEA2-42*	chr5	4.00E-47
ScLEA2-12	chr2	*ScLEA2-20*	chr2	3.00E-11
ScLEA2-20	chr2	*ScLEA2-31*	chr4	2.00E-56
ScLEA2-10	chr2	*ScLEA2-31*	chr4	5.00E-13
ScLEA2-28	chr3	*ScLEA2-34*	chr4	1.00E-48
ScLEA2-26	chr3	*ScLEA2-51*	chr7	4.00E-19
ScLEA1-7	chr4	*ScLEA1-10*	chr4	2.00E-25
ScLEA1-4	chr4	*ScLEA1-5*	chr4	8.00E-51
ScLEA1-9	chr4	*ScLEA1-10*	chr4	1.00E-20
ScLEA1-6	chr4	*ScLEA1-9*	chr4	1.00E-28
ScLEA2-31	chr4	*ScLEA2-40*	chr5	2.00E-126
ScLEA2-33	chr4	*ScLEA2-42*	chr5	6.00E-78
ScLEA2-34	chr4	*ScLEA2-41*	chr5	5.00E-97
ScDehydrin-3	chr4	*ScDehydrin-18*	chr6	1.00E-14
ScLEA2-39	chr5	*ScLEA2-55*	chr7	5.00E-17
ScSMP-1	chr5	*ScSMP-4*	chr7	4.00E-16
