# Supplementary material for: Genome-wide identification and expression analysis of late embryogenesis abundant protein-encoding genes in rye (Secale cereale L.)
Source: PLoS One. 2021 Apr 8;16(4):e0249757. doi: 10.1371/journal.pone.0249757 (PMC8031920; doi:10.1371/journal.pone.0249757)
Supplement: Attachment — Submitted filename: Response to Reviewers.docx [file pone.0249757.s010.docx]

Dear Reviewer 1，

Thanks for your comments on our paper. We have revised our paper according to your comments:

1. qPCR analysis: sampling strategy and statistical analysis must be described in the M&Ms.

**Answer**: Sampling strategy and statistical analysis have been added in the M&Ms.

2. Results: avoid literature citation in the Results section.

**Answer:** References cited in the results section, transferred to the introduction or methods section, or deleted.

3. Other minor suggestions are marked in the edited manuscript.

**Answer**: Adoption of reviewer suggestions.
